# Dynamic hetero-metallic bondings visualized by sequential atom imaging

**DOI:** 10.1038/s41467-022-30533-y

**Published:** 2022-05-27

**Authors:** Minori Inazu, Yuji Akada, Takane Imaoka, Yoko Hayashi, Chinami Takashima, Hiromi Nakai, Kimihisa Yamamoto

**Affiliations:** 1 Laboratory for Chemistry and Life Science, Tokyo Institute of Technology, Yokohama, 226-8503 Japan; 2 ERATO-JST Yamamoto Atom Hybrid Project, Tokyo Institute of Technology, Yokohama, 226-8503 Japan; 3 Department of Chemistry and Biochemistry, School of Advanced Science and Engineering, Waseda University, Tokyo, 169-8555 Japan; 4 Waseda Research Institute for Science and Engineering, Waseda University, Tokyo, 169-8555 Japan; 5 ESICB, Kyoto University, Kyotodaigaku-Katsura, Nishikyo-ku, Kyoto, 615-8520 Japan

**Keywords:** Chemical physics, Nanoparticles, Nanoscale materials

## Abstract

Traditionally, chemistry has been developed to obtain thermodynamically stable and isolable compounds such as molecules and solids by chemical reactions. However, recent developments in computational chemistry have placed increased importance on studying the dynamic assembly and disassembly of atoms and molecules formed in situ. This study directly visualizes the formation and dissociation dynamics of labile dimers and trimers at atomic resolution with elemental identification. The video recordings of many homo- and hetero-metallic dimers are carried out by combining scanning transmission electron microscopy (STEM) with elemental identification based on the Z-contrast principle. Even short-lived molecules with low probability of existence such as AuAg, AgCu, and AuAgCu are directly visualized as a result of identifying moving atoms at low electron doses.

## Abstract (web summary)

The dynamic assembly and disassembly of atoms and molecules is challenging to characterize in real time, with atomic resolution and elemental identification. Here, the authors report direct observation of more than twenty homo and hetero-metallic compounds, including labile Ag-Cu dimers and Au-Ag-Cu trimers.

## Introduction

Interatomic bond formation or dissociation is the essence of chemistry. The knowledge about chemical bonds derived from the structures and reactions of many chemicals has been fundamental to organic, inorganic, and coordination chemistry. However, cutting-edge chemistry and material science target not only traditional isolable compounds but also the dynamic assembly and disassembly of atoms and molecules formed in situ that are difficult to observe directly. The latter is justified only based on kinetic data, spectral data, and microscopic analysis. An example is the metastable nanostructures of metals. In particular, metal clusters and subnanoparticles are a group of materials dissimilar to nanoparticles or bulk materials in that their properties vary significantly depending on the number of atoms and the composition of the metal atoms^1–4^. With the exception of stable ligand-protected clusters^5,6^, the isolation and structure determination of metal clusters is challenging. Therefore, the observation and characterization of moving metal atoms in fluxional metal clusters^7–10^ predicted by computational chemistry make this topic a frontier in chemistry and nanoscience. More recently, isolated atoms and their aggregates (clusters) that evolve through reconfiguration during the progress of catalysis, are considered as key active species^11–14^. In this context, the importance of the atomic-scale dynamic structural chemistry of metal assemblies has been increasing.

As mentioned, investigating the bonds of dynamic compounds could facilitate significant advances in these new research areas, but this necessitates the introduction of new methodologies. Conventional structural analyses based on X-ray diffraction (XRD)^15,16^ or absorption (XAFS)^17,18^ only provide average structural information; thus, they are ineffective in studying the above-mentioned dynamic compounds. As a new approach, electron microscopy could be one such powerful tool. In the 1980s, electron microscopes have enabled visualizing the motion of atoms on the surface of solids^19–23^. Furthermore, recent advances in aberration-corrected electron microscopy^24^ and sensors (CCD and CMOS) have allowed the observations with high spatial and temporal resolution at the atomic scale and ms time scale. For example, imaging of atoms^25,26^ and their aggregates^27–30^ supported on carbon or metal oxides yields insights into atom-support interactions^31–33^. Besides the static snapshots, real-time videos of moving atoms^34–36^ and molecules^37^ bear witness to many critical moments such as crystallization^38^, bond formation^39^, and fluxional isomerizations of clusters^27–30,40–44^.

The next challenge is to discover new substances that have never been observed before and to elucidate their structure and properties via visual observation. Thus, this study seeks to observe homometallic and heterometallic bond formation and dissociation in atom dynamics. In particular, there is a lot of untapped heterometallic bonding between immiscible elements because their pure compounds are unavailable at both the bulk and atomic scale. Nevertheless, such heterometallic bonds should not be ignored, as they could provide important information regarding the design of sub-nano alloy particles (atom-hybridization)^45–47^ or high-entropy alloys^48^.

Here, we show the direct visualizations of the formation and dissociation dynamics of labile dimers and trimers at atomic resolution with elemental identification. To study the transient behavior of such heterometallic compounds during observation, the identification of elements in the continuous images is a prerequisite. Energy-dispersive X-ray spectroscopy (EDS)^49–51^ and electron energy-loss spectroscopy (EELS)^52–55^ are commonly used as elemental analysis techniques in combination with atomic-resolution transmission electron microscopy. However, the application of these methods to single-atom analysis requires a very high current density that results in severe knock-on damage^56–60^. Therefore, neither of these methods is suitable for the study of moving atoms. Instead, accurate elemental identification of atoms under low current density based on the Z-contrast principle of annular darkfield scanning transmission electron microscope (ADF-STEM) in combination with video-based atom-tracking is proposed in this study.

## Results and discussion

### Preparations of hetero-atomic dispersions

**Fig. 1:**
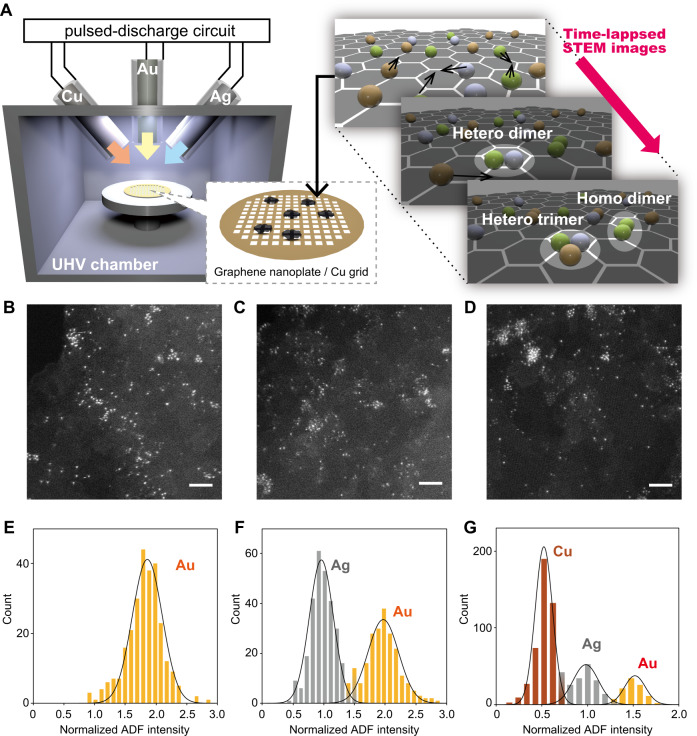
A schematic illustration of the ADF-STEM images and the result of elemental identification in various samples. **A** A conceptual diagram of the process from sample preparation to STEM observation. During the observation, Au (yellow), Ag (gray), and Cu (brown) atoms move around randomly on the graphene as indicated by the arrows, forming not only homodimers but also heterodimers and heterotrimers. In this experiment, the atoms deposited on graphene by APD are captured using STEM. **B**–**D** ADF-STEM images of Au (**B**), Au/Ag bimetallic (**C**), Au/Ag/Cu trimetallic dispersion (**D**). Scale bar: 2 nm. **E**–**G** Histogram of the ADF intensity in monometallic Au (**E**), bimetallic Au/Ag (**F**), and trimetallic Au/Ag/Cu (**G**).

Specimens for the observation of atom dynamics were prepared as follows (Fig. 1A). First, a suspension of graphene nanoplatelets in methanol was cast on a Cu microgrid mesh. Transition metal atoms (Au, Ag, Cu, Pt, or Pd) were then deposited onto the graphene nanoplatelets using an arc plasma-deposition (APD) method. The amount of atoms deposited on graphene depends on several parameters such as capacitor capacitance, applied voltage, and the number of discharge pulses. The atomic-resolution STEM observations of samples prepared under various conditions showed that large nanoparticles were produced and surface density increased as the number of discharge pulses and capacitor capacitance were increased (Supplementary Fig. 1A). In this experiment, in order to provide efficiently isolated single-atom or very small clusters (dimer or trimer), these samples were prepared so that the total deposition amount was set to *ca*. 0.05–0.015 monolayers based on the deposition ratio calculated using the quartz crystal microbalance (QCM) method (see “Methods”). These atoms tend to collect at the edges of the graphene sheets, indicating that the dangling bonds or partial defects of the graphene strongly affected the atomic dispersion. Thus, the high-magnification observation of atoms and their dynamics was conducted on flat areas of the graphene surfaces.

In addition to the homometallic dispersions, heterometallic dispersions (Au–Ag, Au–Cu, Ag–Cu, and Au–Ag–Cu) on graphene-modified Cu grids were available using two or three kinds of APD targets. The number of discharges for each element was optimized based on the deposition rates for each element (Supplementary Fig. 1 and Supplementary Note 1) to equalize the number of atoms deposited. An ADF-STEM image of Au–Ag dispersion is shown in Fig. 1C, illustrating the coexistence of atoms with different brightness levels.

### Identification of moving atoms

Elemental identification, which is available with real-time tracking of the moving atoms, is essential to identify the instances of labile bond formation and dissociation between different elements. The present approach based on the Z-contrast principle of ADF-STEM imaging has an advantage for the observation of atoms under low electron dose. The *Z*-contrast principle is based on the mean value of electron scattering intensity (*I*), which is proportional to the square of the elastic scattering amplitude (*f*). Because *f* is approximately proportional to the atomic number (*Z*) for high-angle scattered electrons, *I* is, in principle, proportional to *Z*^260^. Although the *Z*-contrast-based approach has been used for structural analysis of robust crystalline materials^61^, the application to moving atoms has not yet been reported.

Quantifying the scattering intensity (*I*) based on the brightness of ADF-STEM imaging requires the preprocessing of the experimental images, as shown in Supplementary Fig. 2. First, the background over the entire image was flattened to remove the scattering intensity, mainly due to the graphene substrate. Then, the shot noise was reduced by the application of a mean filter. The parameters used for image preprocessing are shown in the Method section. Note that careful image preprocessing is required to extract the atomic information, as described in Supplementary Fig. 3. Given that the maximum intensity value for each atom extracted from the processed STEM is used for the assignment of an element, insufficient noise reduction would increase invalid assignments. Conversely, excess preprocessing may lead to a loss of information. In addition, it should be noted that an accurate atomic scattering intensity value is only available when the observed atoms do not overlap with the other atom. Because the overlapping of atoms results in multiplying the brightness in a STEM image, the double- intensity values were excluded from the analysis.

Every histogram of the scattering intensity (*I*) for monometallic Au, Ag, or Cu yielded a single peak (Fig. 1E and Supplementary Fig. 4). Based on the principle of electron scattering, the intensity (*I*) should exhibit a Poisson distribution. If the number of electrons is sufficiently large, the Poisson distribution can be approximated by a normal distribution. In fact, each histogram was analogous to a normal distribution, and the standard deviation was *ca*. 20% of the mean value. An exact reason for this deviation is not known, but several reasons underlying the intrinsic and technical factors must be complexed. Basically, a stochastic distribution (Poisson distribution) for the scattering intensity of electrons is generated due to the particle nature of electrons. Another intrinsic factor is the thermal diffuse scattering (TDS)^62^, the contribution of which increases as the ADF detector is placed at a higher angle. Furthermore, the contribution of TDS to the image is dependent on the Debye–Waller factors (also called the thermal or B factor) of the atoms. The Debye–Waller factor is, in principle, different for atoms at different positions. Unfortunately, there is no suitable model for determining the Debye–Waller factors of single atoms on graphene. Other technical factors may also cause intensity deviations. For example, the motion of an atom during the observation causes a blurring of the atomic image. In a scanning microscope, the probe scans each line sequentially. Depending on the beam scanning and the timing of atom movement, atoms may completely disappear for a moment, or appear twice as previously reported^25^. Even without such extreme cases, the observed intensity of an atom becomes weaker as the probe misses the center. In addition, defocusing or incomplete background subtraction could be another reason for intensity deviations. ADF intensity errors due to defocusing are more pronounced in a spherical aberration-corrected STEM with a narrow depth of focus^63^. The 20% standard deviation mentioned above can be considered to include all of these factors.

Although the observed ADF intensities varied by about 20%, the discrimination of Au, Ag, and Cu atoms based on the Z-contrast of a one-frame image is reliable enough. The histograms of the scattering intensity (*I*) (Fig. 1F, G) exhibited separate peaks for mixed bimetallic or trimetallic samples (Au–Ag, Au–Pd, Au–Cu, and Au–Ag–Cu). Moreover, the obtained histograms were subjected to multi-curve fitting by normal distributions to calculate the intensity ratio of each element and the accuracy of the atom discrimination in the heterometallic dispersions. For the Au–Ag pair, the overall accuracy of the atom discrimination was 98.7%. Furthermore, in the case of the Au–Cu pair, the overall accuracy of atom discrimination was 99.9%. Other pairs, Ag–Cu, Au–Cu, Pt–Pd, Pt–Ag, and Au–Pd, were also discriminated at almost the same level (Supplementary Fig. 5).

As mentioned above, the present approach provides highly accurate discrimination between the different periodic elements. Detecting identification errors, which exist in up to 2% of cases, and improving the method to be more reliable will further expand the range of applicable elements. If the electron scattering intensity distribution is a Poisson distribution, then integrating the values should effectively narrow the distribution. However, as isolated atoms and dimers move, a simple image summation, as is performed in atomic columnar visualization of the crystal lattice, is unavailable. To deal with this problem, we tracked the atoms moving between frames by image analysis and integrated the scattering intensities (*I*) for ten frames along with the tracking data. Accordingly, we succeeded in reducing the distribution to about 12% (Supplementary Fig. 6). This reduction in the standard deviation leads to an improvement in the accuracy of elemental identification. Furthermore, by applying this method to the movies of heterometallic dimers and trimers, it is possible to completely discriminate atoms of different periodic elements (Supplementary Fig. 7).

**Fig. 2:**
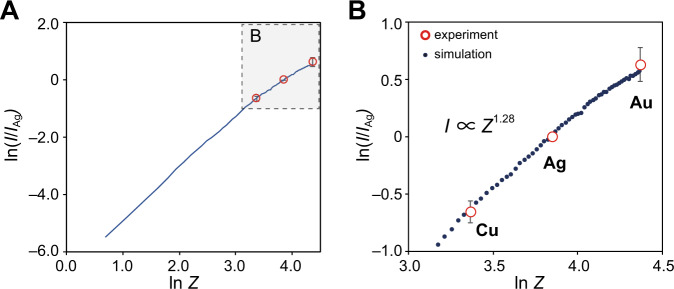
Dependence of the atomic number (*Z*) of the annular-dark-field (ADF) intensity of single atoms based on simulation and experiment. **A** The logarithmic plot of *Z* versus relative ADF intensity (*I/I*_Ag_*)* was created based on the simulation (black dot) and the experiment (red circle). **B** A figure showing Panel A extended to the entire atomic number range. The ADF intensity of each atom (*I*) is normalized to that of an Ag atom (*I*_Ag_*)*. The error bars represent the averages and the standard deviations of the experimental data. The elements in the simulation ranged from hydrogen (*Z* = 1) to gold (*Z* = 79).

The assignments to each element were further verified by comparing the experimental *Z*-contrast with the corresponding simulation. The peak-top values of the normal distribution derived from the curve fitting of the histograms were taken as experimental single-atom ADF intensities (*I*) for each element. The comparison of the experimentally determined *I* with the simulated *I* (Supplementary Note 3) shows good agreement, indicating that the elemental identification is reasonable (Fig. 2A). Here, both the experimental and simulated *I* are normalized to 1 at Ag. The logarithmic plot of *Z* versus the experimental and simulated *I* was both linear with the slopes of 1.28 and 1.20, respectively (Fig. 2A). As already discussed, *I* is, in principle^61,62,64^, proportional to *Z*^2^ in high-angle ADF-STEM imaging. However, the experimental *Z*-contrast often deviates from this law, and the power order α, where *Z*^α^, in general, takes an intermediate value between 1 and 2. The results of α in this study were no exception, but they are much smaller value than would be expected from common understanding.

To understand the results in more detail, STEM simulations were carried out for every single atom, ranging from hydrogen (*Z* = 1) to gold (*Z* = 79) (Fig. 2B and Supplementary Fig. 9). The logarithmic plot of *Z* versus *I* based on the simulation indicated the exponent, *n*(*Z*), corresponding to the increment of *I* for each element with respect to *I* for hydrogen (*Z* = 1). The calculated *n*(*Z*) values were distributed from 1.9 to 1.7, which was approximately equivalent to the previous result by Treacy^62^. Meanwhile, the slope of the plot only for the experimentally observed elements (Au, Ag, Cu) was only 1.20, which agrees well with the experimental value (*α* = 1.28). The reason for the smaller α value than *n*(*Z*) is the deviation from the power-law for high *Z* atoms, which was previously discussed by Kimoto et al.^64^. From the experimental and the simulated results, it was concluded that the ADF intensity values of atoms after the appropriate image preprocessing provide information that allows the discrimination of elements.

### Heterometallic bond formation and cleavage

The metal atoms move around randomly on graphene (Supplementary Movie 1). This motion is caused by a combination of the direct process due to elastic collisions of electron beams, the indirect process with excitation due to inelastic collisions, and thermal energy. The main factor depends on the activation barrier required for the movement, the temperature, the acceleration voltage of the electron beam, and the current density^59^. However, it should be noted that the increase of the local temperature in the observation area was calculated to be negligible because of the high thermal conductivity of graphitic carbon^65,66^.

**Fig. 3:**
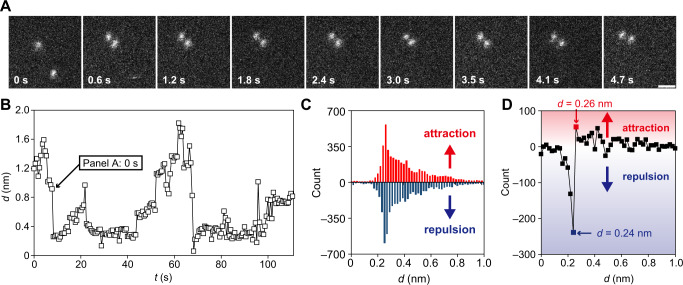
STEM movie analysis in Au dimers. **A** Snapshot images of the two gold atoms producing an Au–Au dimer. Scale bar: 0.5 nm. **B** Temporal change of the interatomic distance d in the Au–Au dimer. The arrow corresponds to the 0-second moment in (**A**). **C** Evaluation of the direction of the force in the Au–Au dimer. A positive and negative count expresses the repulsion (red) and the attraction (blue), respectively. **D** The count of the attraction side of (**C**) subtracting the count of the repulsion side of the same figure. This figure indicates that the attraction and repulsion forces switch at d between 0.24 and 0.26 nm.

Atoms in close proximity to each other are frequently bound to each other under the STEM observation. For example, a trajectory of a pair of gold atoms is shown in Supplementary Fig. 9A. When the approaching gold atoms reach a certain distance (*ca*. 0.3–0.4 nm), they suddenly form a bond, and the bond distance of about 0.26 nm is kept for a few seconds (Fig. 3A, Supplementary Figs. 10A, and 14). As shown in Supplementary Fig. 10B, the observed average lifetime of the gold dimer was approximately independent of the electron beam acceleration voltage (40–80 kV). This result suggests that the driving force for bond breaking is mainly thermal rather than electron beam under low acceleration voltage (80 kV) in our experimental conditions. This idea is consistent with the semi-quantitative interpretation that the maximum energy transferred by an 80 keV electron in an elastic collision with a gold atom is 0.9 eV^59^, which does not provide the energy (*ca*. 2 eV)^67^ required bond breaking of a gold dimer (Supplementary Note 2).

The formation and dissociation of the gold dimer are reversible. The histogram of the projected distance (*d*) between two atoms indicated that Au dimers have a high frequency of values around *d* = 0.26 nm (Supplementary Fig. 15E). This observation implies that the bonding distance of Au dimer is 0.26 nm. Based on this idea, projected images observed at shorter distances (*d* < 0.26 nm) mean that one atom is partially mounted on the other atom, while projected images observed at longer distances (*d* > 0.26 nm) mean that the interatomic bond is broken. Furthermore, the trajectory analysis of Au dimers strengthens the idea that the most frequently observed distance is the equilibrium bond length. We define the change in *d* as Δ*d* = *d’* − *d* where the observed *d* in one frame becomes *d*’ in the next frame. Figure 3C shows the frequency of negative (blue) and positive (red) values of Δ*d* at each *d*. When *d* > 0.26 nm, Δ*d* tends to be positive, while for *d* < 0.24 nm, Δ*d* tends to be negative. This result means that the attraction and repulsion forces switch between 0.24 and 0.26 nm. The boundary is approximately consistent with the calculated^67–69^ and experimental (0.247 nm)^70^ bond distances of Au_2_ in a vacuum. Due to the weak interaction between Au atoms and graphene nearly equivalent to van der Waals interaction, the calculated bond distance of Au dimers on graphene (0.250–0.257 nm)^71^ is also within the range of the present experiment.

**Fig. 4:**
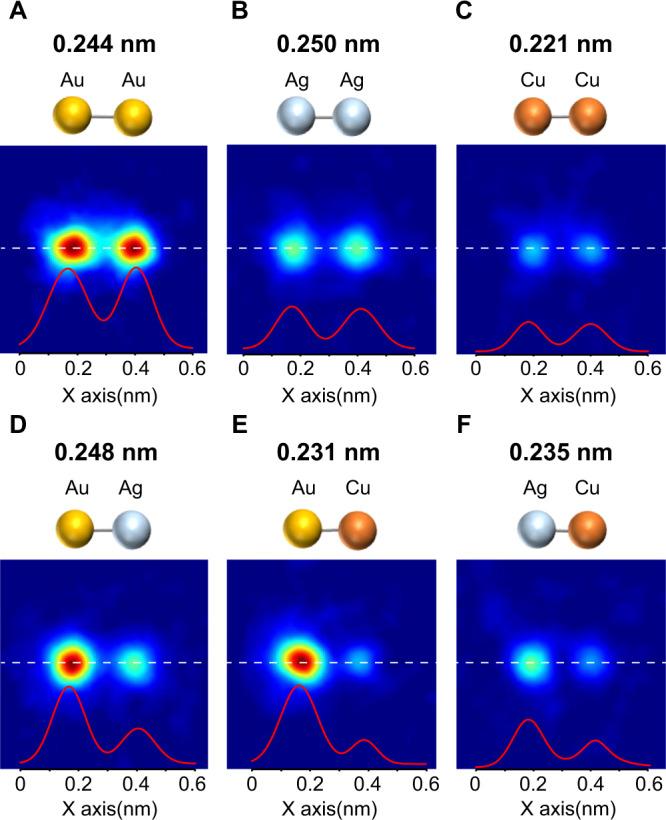
ADF-STEM averaged images of homometallic and heterometallic dimers. **A**–**F** Most stable structure by DFT calculation (upper part) and the corresponding average STEM image at the calculated equilibrium interatomic distance (lower part). The average images are composed of multiple snapshots during the movie recording. Each image (**A**–**F**) corresponds to Au–Au, Ag–Ag, Cu–Cu, Au–Ag, Au–Cu, and Ag–Cu, respectively. Each red line in the image represents the cross section of the ADF intensity on the line passing through the center of both atoms.

Similar proximity behaviors were observed for other atomic pairs of the same element (Ag–Ag and Cu–Cu) and different elements (Au–Ag, Au–Cu, and Ag–Cu) as shown in Supplementary Movies 2–4. The elements of atoms were identified using the above-mentioned *Z*-contrast analysis of STEM (Supplementary Fig. 8). Not only the bond formations but also the cleavage of the dimers into separate single atoms were observed. Although the observation of the moment of cleavage and bonding of metal atoms of the same species has been recently visualized^72^, to the best of our knowledge, this is the first example that such a moment of heterometallic bond cleavage and formation has been directly observed on video. The tracking analysis also allows the production of average images of homo and heterodimers with high *s/n* quality by extracting the images at the bonding distance from the entire movie (Fig. 4). Previous calculations have confirmed that graphene has little effect on the bonds between these group-11 elements since the interactions of these atoms (Au, Ag, Cu) with graphene are very weak^73^. In fact, our STEM observations have directly captured the moment when the dimeric structure on graphene is consistent with the stable structure in a vacuum.

**Fig. 5:**
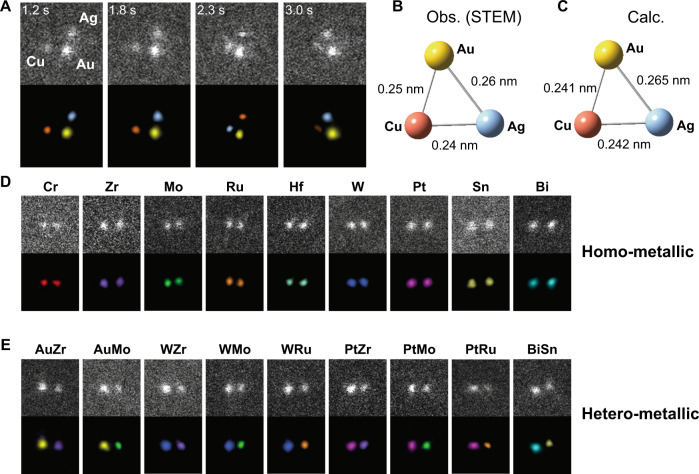
STEM observation of the Au–Ag–Cu trimer and various dimers. **A** ADF-STEM snapshot images of the Au–Ag–Cu trimer. The lower images are created by coloring the upper images. The actual size of one side of each frame is 1.22 nm. **B** The structure of Au–Ag–Cu trimer with averaged bond distances of the snapshots in the panel (**A**). **C** The optimized structure of Au–Ag–Cu trimer in vacuum by the calculation. **D** ADF-STEM images of homometallic dimers (Cr_2_, Zr_2_, Mo_2_, Ru_2_, Hf_2_, W_2_, Pt_2_, Sn_2_, and Bi_2_). **E** ADF-STEM images of the heterometallic dimers (AuZr, AuMo, WZr, WMo, WRu, PtZr, PtMo, PtRu, and BiSn). For (**D**, **E**), the upper images show the original data, whereas the lower images show the ones colored by the element based on the electron scattering intensity values.

Most strikingly, we have also succeeded in capturing the heterometallic tri-atomic cluster molecule AuAgCu (Fig. 5A). The brightness of the three atoms that comprise the tri-atom cluster differs from each other. In comparison with the elemental dependence of the ADF intensity described in the previous section, each atom is assigned to Au, Ag, and Cu. Also, the X-ray photoelectron spectra (XPS) of the obtained whole samples confirmed the respective elements (Supplementary Fig. 11 and Supplementary Note 5). To the best of our knowledge, no such molecule has ever been observed in the previous research, including studies based on the use of gas-phase synthesis methods. The calculated bond distances for AuAgCu are 0.265 nm, 0.241 nm, and 0.242 nm for Au–Ag, Au–Cu, and Ag–Cu, respectively (Fig. 5C). Although we could not obtain a snapshot in perfect agreement with the structure by theoretical calculation, the average bond lengths between the elements in the observed structure were in good agreement with the calculation (Fig. 5B, C and Supplementary Note 6). According to the phase diagram, the homogeneous mixing of Au, Ag, and Cu requires high temperatures above 800 °C^74^ reflecting the poor compatibility of Ag and Cu^75^. It is remarkable that the combination of dynamic electron microscopy and elemental identification enables the structural characterization of molecules and clusters that cannot be produced in bulk to the nanoscale.

In addition, the method used in this study was applied to a large variety of metallic elements, including early transition metals and typical metals. In fact, we have succeeded in observing not only homometallic dimers ranging from Cr to Bi (Fig. 5D and Supplementary Note 7) but also heterometallic dimers as movies (Fig. 5E and Supplementary Movie 5). The histograms of the projected distances shown in Supplementary Fig. 14 strongly suggest the presence of intermetallic bonding in all dimers. However, unlike the dimers of the 11 elements, the observed projection distances of these base metal dimers tend to be longer than the experimental and calculated bond distances in a vacuum. This fact suggests that these base metal dimers interact strongly with graphene or oxygen, in contrast to the dimers of group-11 elements, where the interaction has little effect on their structure.

In summary, we have succeeded in directly visualizing the AuAgCu cluster molecule by combining the atom-dynamics recording and the *Z*-contrast-based atom identification. Although the identification of elements using STEM *Z*-contrast was only for atomic columns^76,77^, we have succeeded in extending this technique to moving atoms with high precision. The visualization of even a small amount of previously unobservable substance has significance for advancing chemistry that effectively utilizes a small number of these chemical species. For example, the control over increasing the number of unstable chemical species in a reaction allows the design of dynamic single-atom catalysts. This research may contribute to advance new chemistry and materials science such as alloy subnanoparticles and high-entropy alloys.

## Methods

### Sample preparation

Graphene nanoplatelet (GNP, carbon >85 wt.%, Sigma-Aldrich) was ultrasonicated for 3 h in 3 mol L^–1^ HCl aqueous solution to remove metal impurities. After the filtration of this mixture, the residue was washed with ultrapure water (>18 MΩ) three times and subsequently rinsed with methanol. After the filtration, the purified GNP clumps were ground to be as fine as possible and dried at room temperature for few hours in air and finally vacuum-dried for few days at 200 °C. All samples for the STEM observations were prepared by a vacuum deposition method using a pulsed arc plasma source (Advance Riko, APS-1) equipped with a metal cylinder target. A methanol dispersion of the purified GNP was cast onto a Cu grid-attached holy carbon mesh, dried thoroughly, and then various atoms were deposited by arc plasma deposition. At a frequency of 1 Hz, an arc pulse with a period of 0.2 ms and a current amplitude of 2 kA was generated. The plasma from the cathode was directed to the graphene nanoplatelets modified on a TEM grid. In all sample preparations, the deposition ratio of each metal element was unified to be roughly 1:1. However, the total deposition amount was adjusted based on the product of the number of discharge pulses and the deposition rate calculated by the QCM method (Supplementary Fig. 1B, C).

### ADF-STEM observation

A transmission electron microscope (JEOL, JEM-ARM200F) equipped with a spherical aberration-corrected probe (CEOS, ASCOR) and cold-FEG was used for the observation at an acceleration voltage of 80 kV unless otherwise noted. The probe current was fixed at 14 pA where the aperture semi-angle was 31 mrad. The inner and outer collection semi-angles used for the annular-dark-field (ADF) imaging were 57 and 226 mrad, respectively. To keep the electron beam irradiation per area constant for the video recording, the image resolution, pixel density, and pixel time (dwell time for each pixel) were set to 128 × 128 pixels, 61.46 pixel nm^–1^ and 10 µs, respectively. The difference between the scan start times of two adjacent frames was *ca*. 0.5–0.6 s.

### Image processing

The ADF-STEM images were processed to extract brightness values of atoms using the functions of an image processing software (Image J^78^). First, we used the function of “subtract background” on the raw images to flatten the background. The “rolling ball radius” parameter was set to 40 pixels. Then the function of “median filter” or “mean filter” (radius:3 pixels) was used to remove the noise. Finally, the function called “Find Maxima” was used to extract the X–Y coordinates and the brightness values of the atoms without the backgrounds. We investigated the effect of these parameters on the resulting STEM images (Supplementary Fig. 3). Careful selection of the image processing conditions is required because the over/under-subtraction of the background and over/under-filtering can cause inaccurate estimations of the brightness value and the X–Y coordination of atoms. In this study, the optimal settings of the image processing are the “rolling ball radius” of more than 20 pixels for “Subtract background” and the radius of less than 5 pixels for “median or mean filter”. Note that we need to care that the optimal values of these parameters differ depending on the image magnification.

### Identification of atoms for mixed bimetallic samples

Prior to the elemental identification, the histograms of ADF intensities of atoms were made for all samples (Fig. 1 and Supplementary Figs. 4 and 5). First, the background-subtracted brightness values were extracted from five independent images recorded at different positions of the samples using the method described in the previous section. A multi-peak curve-fitting analysis based on a Gaussian-type function was suitably applicable to each histogram. If the histograms contain an Ag component, the peak-top value for the Ag component was normalized to 1. From the Ag-normalized ADF intensities of Au and Cu (Fig. 1F and Supplementary Fig. 5D), the peak-top value of Au and Cu were determined to be 1.88 and 0.52, respectively. These values were used for the normalization of samples that did not contain an Ag component (Fig. 1E, Supplementary Figs. 5D and 6C). The simplest way to identify elements is to use the intermediate value of the peak-top value of each ADF intensity as the threshold for identification. For example, to determine Ag and Au, their peak-top values are 1.00 and 1.88, respectively, therefore, the atom with an intensity larger than the threshold of 1.44 is considered to be Au, and the atom with a smaller intensity is considered to be Ag. Based on the histogram of an Au–Ag system (Fig. 1F), the accuracy of the elemental identification (Au or Ag) by single-frame analysis was 98.7%. This incomplete identification is based on the standard deviation of the ADF intensity value (~20% of the peak-top value for every element).

The accuracy was improved to nearly 100% by utilizing the multiframe atom-tracking analysis. A trajectory of each atom from the video analysis (Supplementary Movie 2) allowed the integration of the ADF intensity of each moving atom over a successive video clip (Supplementary Note 3). The atom tracking was carried out using the “TrackMate” plugin^79^ included in the open-source image processing software (Fiji^80^, a distribution of ImageJ). The upper threshold of the distance traveled in the tracking was set to 0.5 nm for the Au sample. A histogram of the integrated ADF intensities for 10 frames showed that their standard deviations reduced to 12–13% of the peak-top values (Supplementary Fig. 6). This multiframe tracking significantly improved the identification between different kinds of atoms with almost 100% accuracy (Supplementary Fig. 7).

### Theoretical calculation

Geometry optimizations were performed using the second-order Møller–Plesset perturbation (MP2) theory^81^ implemented in the Gaussian 09 suite of program^82^. For core electrons of Cu, Ag, and Au, Stuttgart–Dresden (SDD) pseudopotentials^83^ were used. For their corresponding valence orbitals, correlation-consistent basis sets of triple-*ζ* (VTZ) quality^84^ were adopted. Harmonic vibrational frequencies were calculated analytically at the same computational level in order to confirm the optimized structure as a potential minimum.

## Supplementary information

Supplementary Information

Description of Additional Supplementary Information

Supplementary video 1

Supplementary video 2

Supplementary video 3

Supplementary video 4

Supplementary video 5

## Data Availability

The data that support the findings of this study are available within the main text of this article and its Supplementary Information. The raw data of STEM images to produce the figures in the main text have been deposited in the Figshare repository under accession code 10.6084/m9.figshare.19593790. Any other relevant data are available from the corresponding authors upon request.
