# Supplementary material for: Dynamic hetero-metallic bondings visualized by sequential atom imaging
Source: Nat Commun. 2022 May 27;13:2968. doi: 10.1038/s41467-022-30533-y (PMC9142510; doi:10.1038/s41467-022-30533-y)
Supplement: Supplementary file 1 — Supplementary Information [file 41467_2022_30533_MOESM1_ESM.docx]

Supplementary Information:

**Dynamic hetero-metallic bondings visualized by sequential atom imaging**

K. Yamamoto et al.

**Contents**

**1. Supplementary Notes**

**Note 1** Optimization of the Arc-plasma deposition condition.

**Note 2** Estimation of the interaction between gold atoms and graphene.

**Note 3** Elemental identification utilizing the histogram of ADF intensity

**Note 4** STEM image simulations.

**Note 5** Elemental analysis of Au-Ag-Cu trimetallic dispersion.

**Note 6** An effect of graphene based on the theoretical calculations.

**Note 7** Effect of oxygen-functional groups on graphene based on the theoretical calculations.

**Note 8** Procedure for coloring images of metal dimers.

**2. Supplementary Figures**

**Fig. 1** Optimization of the arc-plasma deposition (APD) conditions for the sample preparation.

**Fig. 2** A series of image processing steps and the resulting ADF-STEM image.

**Fig. 3** Effect of the image processing conditions on the ADF intensity.

**Fig. 4** ADF-STEM observation and analysis in monometallic dispersion.

**Fig. 5** Atomic identification for different combinations of elements.

**Fig. 6** Atom-tracking analysis of Au monometallic sample for the reduction of the standard deviation of ADF intensity.

**Fig. 7** Elemental identification of moving atoms using the atom tracking.

**Fig. 8** Analysis of the width of the peaks in the histogram of ADF intensity.

**Fig. 9** Relationship between ADF-STEM condition and the ADF intensities.

**Fig. 10** Estimation of the driving force for the bond-cleavage of an Au dimer based on evaluation of the bond lifetime.

**Fig. 11** XPS of Au-Ag-Cu trimetallic dispersion.

**Fig. 12** Estimation of the thickness of a graphene sheet based on the ADF intensity.

**Fig. 13** Visualization of a graphene structure, and the trajectories of single gold atom on the graphene recorded by ADF-STEM observation.

**Fig. 14** Temporal changes of interatomic distance between two Au atoms observed at different sites.

**Fig. 15** Histograms of projected interatomic distance in various homometallic dimers and hetero-metallic dimers

**Fig. 16** A procedure for coloring an ADF-STEM images of a heterometallic dimer.

**Fig. 17** Optimized structures of model of graphene which have zigzag and armchair edges.

**Fig. 18.** Optimized structures of graphenes with (a) zigzag and (b) armchair edges, which are modified with epoxy group.

**Fig. 19.** Optimized structures of graphenes with (a) zigzag and (b) armchair edges, which are modified with hydroxy group.

**Fig. 20.** Optimized structures of Au dimers on graphenes with (a) zigzag and (b) armchair edges.

**Fig. 21.** Optimized structures of Au dimers on graphenes with (a) zigzag and (b) armchair edges, which are modified with epoxy group.

**Fig. 22.** Optimized structures of Au dimers on graphenes with (a) zigzag and (b) armchair edges, which are modified with hydroxy group.

**3. Supplementary Tables**

**Table 1** Calculated bond distances [Å] of AuAgCu trimer on different graphene models and in vacuum.

**Table 2** Calculated bond distances of Au dimer. The deviations from the results without considering oxygen-functional groups and graphene are shown in parentheses and square brackets, respectively.

**4. Supplementary References**

**1. Supplementary Notes**

**Supplementary Note 1. Optimization of the Arc-plasma deposition condition.**

For the preparation of observation samples that provide efficiently isolated metal dimers or very small clusters, the amount of metal deposition was optimized by investigating the correlation between the number of pulses of APD, the capacitor capacity and the dispersion of atoms on graphene (Fig. S1A). As shown in Fig. S1B-C, the QCM measurement can determine the deposition rate (QCM rate) by detecting the amount of metal deposited and its frequency change. However, the deposition rate is the mass deposited per pulse divided by the atomic mass of the element, and they are dimensionless numbers representing the number of moles of atoms deposited per pulse. The deposition rate for each element can be multiplied by the number of pulses for each element to estimate the total amount of deposition, and so this value was set to about 0.05-0.015. In addition to the deposition rate, the optimum applied voltages and the capacitor capacitance differ depending on the kinds of elements, so the conditions were adjusted to obtain the appropriate deposition rate in this experiment (Supplementary Table. 1).

**Supplementary Note 2. Estimation of the interaction between gold atoms and graphene.**

The graphene nanoplatelets used in this study are few-layer graphene flakes with an average of 5-7 atomic layers. The thicker graphene layers are not suitable for observing atomic images. Nevertheless, rather thin regions such as monolayers, bilayers as well as trilayers are found near the edge (Fig. S11). All images were acquired in the field of view of these thin atomic layers.

For example, a gold atom on a graphene sheet, the movement of atoms (bright spots) was observed in the field of view of 2.08 nm square at intervals of about 0.5 seconds (Fig. S12). The atoms were always moving randomly, but sometimes they stayed at the same site between successive frames. The images of the respective frames are very noisy; therefore, the structure of graphene could not be observed. However, combining the images of 10 consecutive frames allowed us to clearly visualize the structure of graphene (Fig. S12A). This result confirms that the motion of graphene is negligible compared to the motion of the gold atoms and that the electron beam does not degrade the structure of a graphene sheet.

In the actual observation, the images shown in the lower part of Fig.S12C were captured continuously for 12 seconds. Then, the images after the Au atom moving out of the field of view was taken continuously over 10 frames, which were combined into a single image (Fig. S12A). The lattice of graphene is observed in Fig. S12A, and the regular hexagonal shape in the FFT image (Fig.12B) indicates that the sample plane is perpendicular to the viewing direction. A careful examination of Fig. S12A shows that there is a part of graphene that is slightly brighter than other carbon atoms, which is considered to be an oxygen functional group^1^ on graphene based on comparison with the ADF-STEM simulation image (Fig.12B).

The image shown in the upper part of Fig.S12C is a composite of the lower image of Fig.S12C and the image of Fig.S12A. These composite images indicate that the Au atoms are often located near this oxygen functional group, which is consistent with the following discussion. The interaction between gold atoms and ideal graphene is very weak (Δ*E* < 0.1 eV)^2,3^. Therefore, it is natural to assume that the impact of the elastic scattering of 80 keV electrons and the thermal driving force at room temperature would easily slide over the graphene surface, making it impossible to capture static snapshots of these atoms. Our observation is that the atoms are in random motion with repeated instantaneous movement and rest. This is very similar to the previously reported motions of Au and Cu atoms ^4,5^, and it is most appropriate to consider that defect sites are responsible for the fixation of metal atoms, as previously discussed. The graphene used in this study is not explicitly oxygen functionalized, but given the previous discussion, it is likely that oxygen functional groups play a role.

Previous studies suggested that electron beams with energies of tens of keV can manipulate the structure at the level of individual atoms and defects through covalent bond breaking and recombination, *i.e.*, bond rotation^6^ and the migration of impurities in graphene^7^ and knock-on damage under STEM observation. However, the damage by electron beam irradiation to graphene can be significantly reduced under acceleration voltages of 80 kV or lower. Although complete suppressions of sputtering of carbon atoms and the bond rotation are difficult,^8,9^ the imaging of defect-free graphene is readily possible at accelerating voltages of 80 kV as demonstrated by Meyer et al.^10^ With respect to these previous studies, the present behavior of the metal dimer under 80 kV of acceleration voltage is not likely driven by the defect transport on graphene, but rather by the interaction between metal atoms and the displacement of metal atoms. However, metal atoms should not be visible on a defect-free graphene as mentioned above; therefore, a few hydrocarbon contaminants or oxygen functional groups may play an anchoring role in our experiments. However, the effect of this oxygen functional group on the observed metal dimers was not significant based on the study by computational chemistry as described in Supplementary Note 7.

**Supplementary Note 3. Elemental identification utilizing the histogram of ADF intensity**

The elemental identification in ADF-STEM movies was carried out based on the ADF-intensities of atoms as explained in the Methods section. A pair of elements of different periods, such as Au and Ag, are distinguished with high reliability (98.7%) because the difference in the ADF-intensities are substantially greater than the experimental error. However, it should be noted that misidentification may occur once in about 100 frames, and the frequency increases further when the difference in atomic numbers is smaller. To correct this identification error, the ADF intensity of an atom can be evaluated as an average value, including the previous and next frames of the same track, based on the continuous movement of the tracked atom between consecutive frames. In the Au-Ag example (Fig. S8A), there are two tracks corresponding to the two atoms in one video clip. Although the averaged brightness values of Au are distinctly higher than those of Ag, the single-frame brightness is occasionally reversed at a frame (*e.g.* *t* = 40 s in Fig. S8A). Such unexpected errors in brightness is inevitable due to the principle of STEM, but the detection and correction of these errors is possible by the tracking. All atomic identifications in this study were performed with this tracking method to increase the reliability.

To know the discrimination accuracy in case of tracking, examples of the discrimination for Au-M (M = Nb, Ru, Ag, Sn) heterodimers are shown in Fig. S8B. Each data point corresponds to the average of the ratio of ADF intensities (*I*_M_/*I*_Au_) over an entire video clip (consisting of at least 50 frames). A minimum of 50 individual video clips of different dimers were recorded for each M, and the results of the analysis are shown in Fig. S8B. The four period-5 elements shown here all have *I*_M_/*I*_Au_ smaller than 1 and their standard deviations are sufficiently small that M atoms can be clearly distinguished from Au atoms. The similar tracking analysis can be performed for other heterodimers consisting of period-5 and period-6 elements other than Au, and the reliability of the analysis is regarded as sufficiently high.

**Supplementary Note 4. STEM image simulations.**

The STEM image simulations were carried out using Elbis (TEM / STEM image simulation software, https://en.fhelectronopt.com/) Constants for the simulation, such as acceleration voltage, aperture size, spherical aberrations, and detector angles were set to those of the instrument (JEOL, JEM-ARM200F) used for the observation. The acceleration voltage was 80 kV. The inner and outer collection semi-angles used in recording the ADF images were 57 and 226 mrad, respectively; the aperture size and spherical aberration coefficient *Cs* were set to 31 mrad and 0.004 mm, respectively. A simulation image of Au, Ag, and Cu in the same plane was created as a test run, and it was confirmed that elements with higher atomic numbers appeared with higher intensity than those with lower atomic numbers (Fig. S9A).

Moreover, in this study, not only images of single atoms, but also images in which multiple atoms exist in the same plane were simulated. Specifically, we prepared 10 patterns of 10 atoms arranged in a scattered pattern for each element and each substrate (Fig. S9C), and the average brightness of the 100 atoms was calculated. The mean brightness was calculated using the same procedure as in the experiment, and the atomic-derived radiance was extracted through background removal and normalization. The same process as in Fig. 2 was used to create the logarithmic plot of *Z* versus *I* based on the obtained brightness. As a result, a power order α = 1.16 was indicated, which agreed well with that created for the simulation of a single atom (α = 1.20) (Fig. S9D).

**Supplementary Note 5. Elemental analysis of Au-Ag-Cu trimetallic dispersion.**

XPS spectra of the obtained samples confirmed the existence of the respective elements (Au, Ag, Cu) (Fig. S10). The XPS was recorded by a Shimadzu ESCA-3400 with Mg Kα (10 kV, 20 mA). The sample was prepared using the APD method as well as those for STEM observation. However, from the aspect of detection sensitivity, the deposition amount (corresponding to “total” in Supplementary Table 1) of each element was adjusted to 0.1, respectively.

**Supplementary Note 6. An effect of graphene based on the theoretical calculations.**

The effect of AuAgCu interaction with graphene was evaluated by theoretical calculations. We considered both Zigzag and Armchair types of graphene as shown in Fig. S17. Specifically, we compared the optimized structure of AuAgCu in vacuum (without graphene) with those in contact with the different graphene structures.

The structure shown in Fig. 5 in the main text was calculated at the MP2/SDD-VTZ level. However, this level of calculation was not practical due to the computational cost when considering the interaction with graphene. Instead, the calculation level was ωB97X-D/SDD by density functional theory. The basis sets for valence orbitals were reduced to double-*ζ* quality. Instead, the calculation level was reduced to ωB97X-D^11^/SDD-VDZ by density functional theory. The structural optimizations were performed for all nuclear coordinates including graphene.

The distance between metal atoms in the optimized structure is shown in Supplementary Table 1. The elongation and contraction of the bonds with and without graphene are smaller than the spatial resolution of our electron microscope, indicating that the effect of graphene on the AuAgCu structure is negligible.

**Supplementary Note 7. Effect of oxygen-functional groups on graphene based on the theoretical calculations.**

The effect of oxygen-functional groups to the interaction with Au dimer and graphene was evaluated by theoretical calculations. The calculation level was ωB97X-D/SDD-VDZ as in Section S6. Both zigzag and armchair types of graphene were considered. We examined two kinds of oxygen functional groups, namely, epoxy and hydroxy groups as shown in Figs. S18 and S19. We compared the bond lengths of Au dimers on graphene without and with oxygen-functional groups. The optimized structures are shown in Figs. S20-S22. The interatomic distances are listed in Supplementary Table 2. The elongation and contraction of the bond with and without oxygen-functional groups are less than 0.002 Å, indicating that the effect of oxygen-functional groups on Au dimer is minor.

The interatomic distance of Au dimer in vacuum (without graphene) was also computed by the same calculation level, resulting in 2.547 Å. The differences in Au-Au distances with and without graphene are also shown in Supplementary Table 2. The inter atomic distances are shortened by 0.004-0.008 Å by the presence of graphene. As concluded in Section S6, the influence of the graphene is also expected to be small for the bond distance of Au dimer.

**Supplementary Note 8. Procedure for coloring images of metal dimers.**

Colored images of homo- and hetero-metal dimer were generated using ImageJ software by the following procedure as schematically in Fig. S16. Two homemade ImageJ macros were used for this coloring process. The first macro runs the (i) to (v) process automatically. The preprocessed image is duplicated after running (i) resizing, (ii) background subtraction, (iii) filtering, and (iv) extraction of the X-Y coordinates of each atom. In each image, the bright spot of the other atom is removed by cropping using the coordinates of the atom extracted in (iv) in order to leave only one atom of each (v). After running the first macro, editing Look-up Table (LUT) makes each atom colored as we set. Thereafter, brightness and contrast are adjusted. Then, we ran the second macro for the completion. The second macro transforms the images into RGB color, and two images are stacked together. Then, the stack is Z-projected by max intensity of two images. At last, the final image was cropped from 256 × 256 pixels to 150 × 150 pixels.

**2. Supplementary Figures**


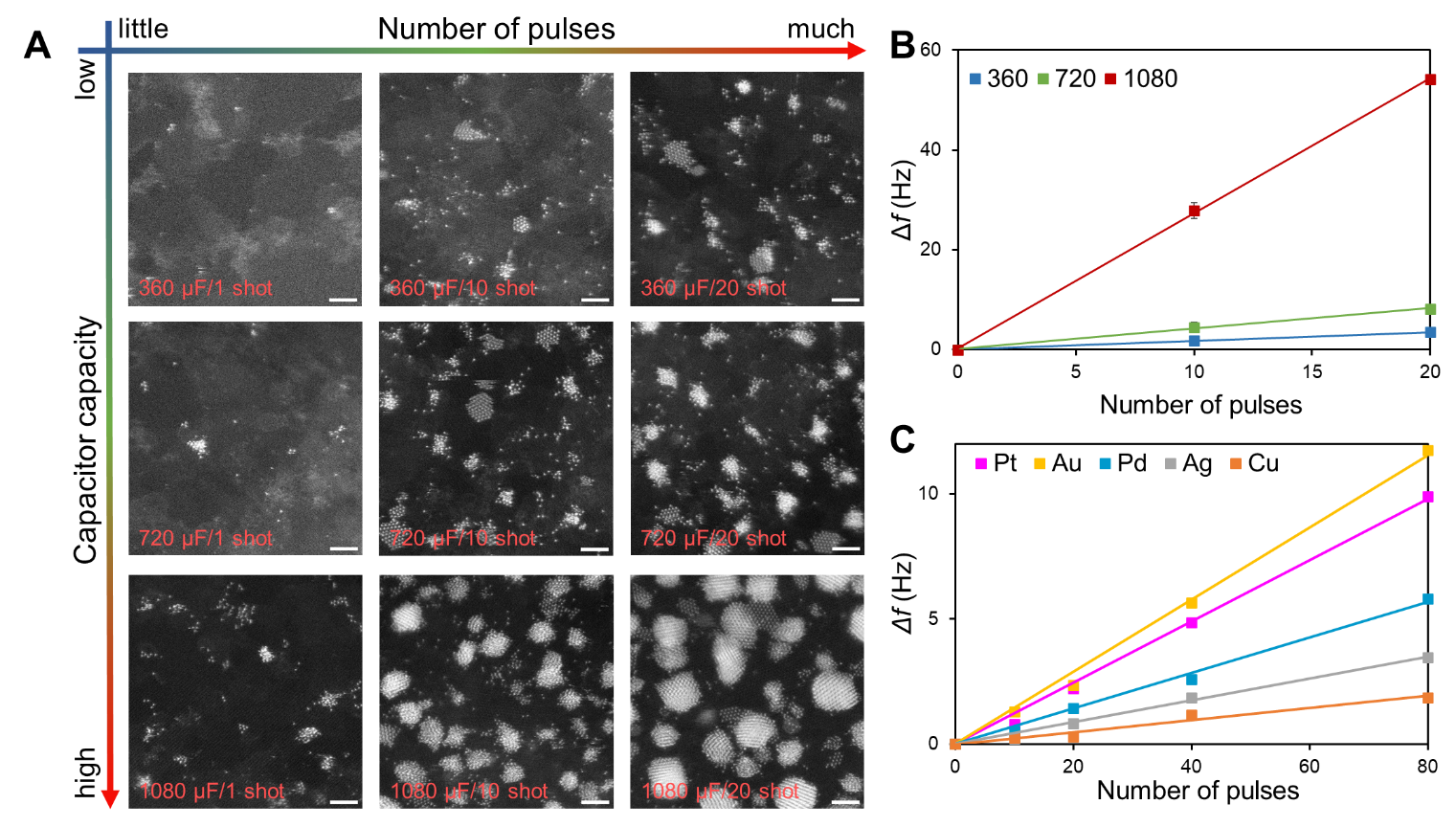


**Supplementary Figure 1.** Optimization of conditions for sample preparation. (A) Comparison of the number of deposited atoms in ADF-STEM images as the number of pulses and capacitor capacity change. The number of pulses and the capacitor capacity was changed in three steps of 1, 10, 20 shots and 360, 720, 1080 µF, respectively. Scale bar is 2 nm. (B) Deposition rate for different capacities (=360, 720, 1080 µF). The frequency changes (Δ*f* [Hz]) of the QCM sensor represent the amount of depositions. The slope of the linear in plot correspond to the deposition rate (C) Comparison of deposition ratio for various elements calculated by plots of metal deposition versus frequency change (Δ*f* [Hz]) in the QCM method.


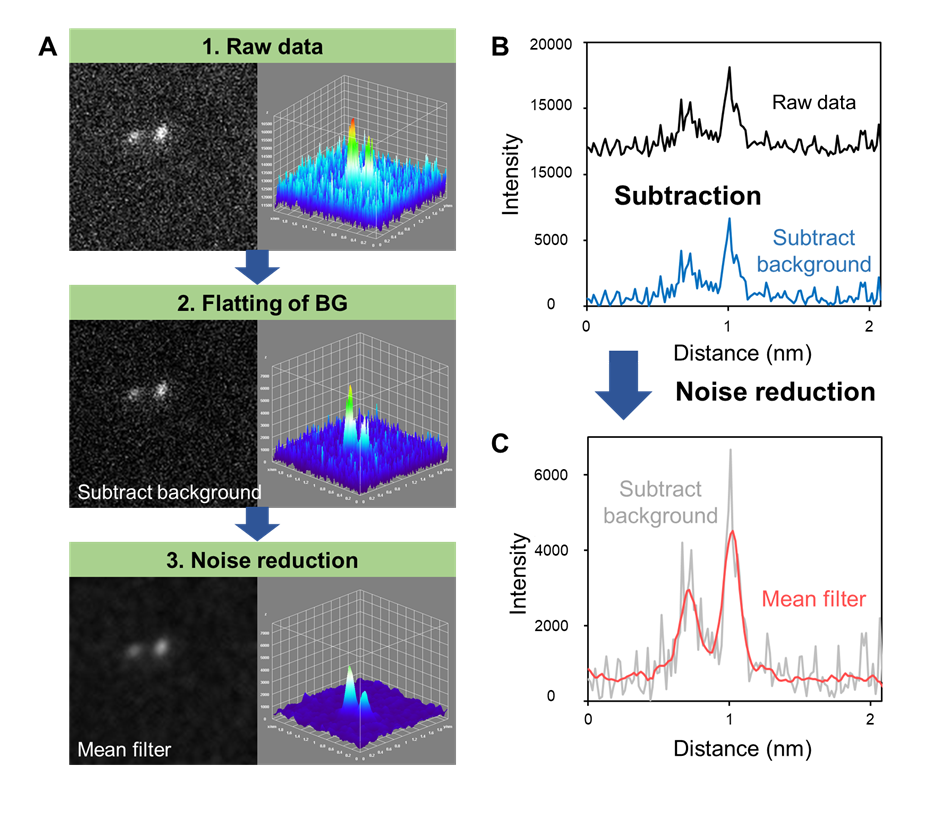


**Supplementary Figure 2.** A series of image processing steps and the resulting ADF-STEM image. (A) Flow chart from raw data to the noise-reduced image. The left images are ADF-STEM images, and the right images are the corresponding 3D surface plot generated by ImageJ software. Each of the three types of images corresponds to an image after each process has been applied. (B) Line plots showing changes in the ADF intensity before and after the background subtraction. (C) Line plots showing changes in the ADF intensity before and after denoising an image with background subtraction.


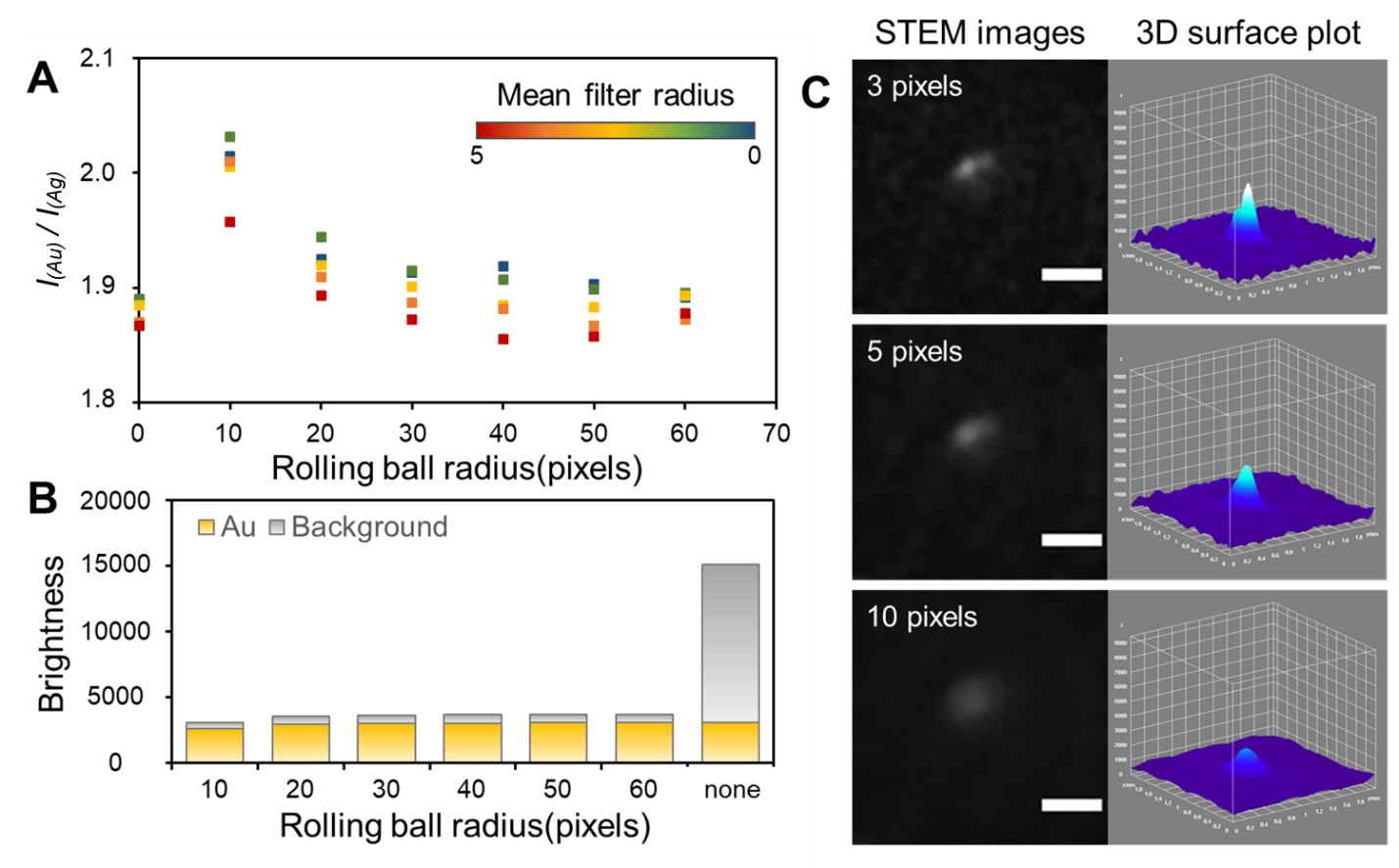


**Supplementary Figure 3.** Effect of the image processing conditions on the ADF intensity. (A) Variation of the ADF intensity ratio of Au and Ag atoms, *I_(Au)_/I_(Ag)_* with changing the parameters in two image processing steps. (B) Change in the ADF intensity of Au single atom extracted from the images after different conditions of the background subtraction. (C) The effect of kernel size for median filter exhibiting the loss of resolution with too large kernel size. Scale bar: 0.5 nm.


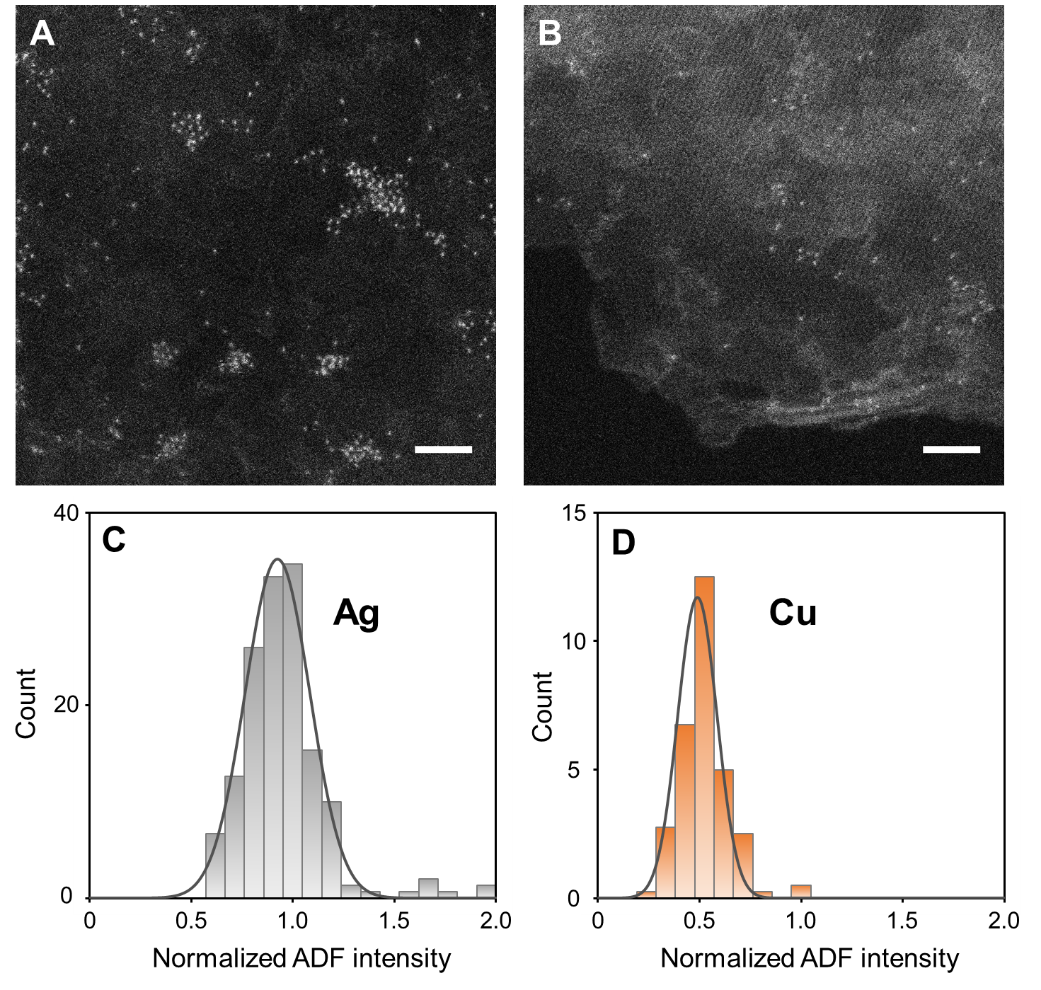


**Supplementary Figure 4.** ADF-STEM observation and analysis in monometallic dispersion. (A,B) ADF-STEM images of Ag (A) and Cu (B) monometallic dispersion. Scale bar: 2 nm. (C, D) Histograms of the normalized ADF intensity in Ag (C) and Cu (D) monometallic dispersion.


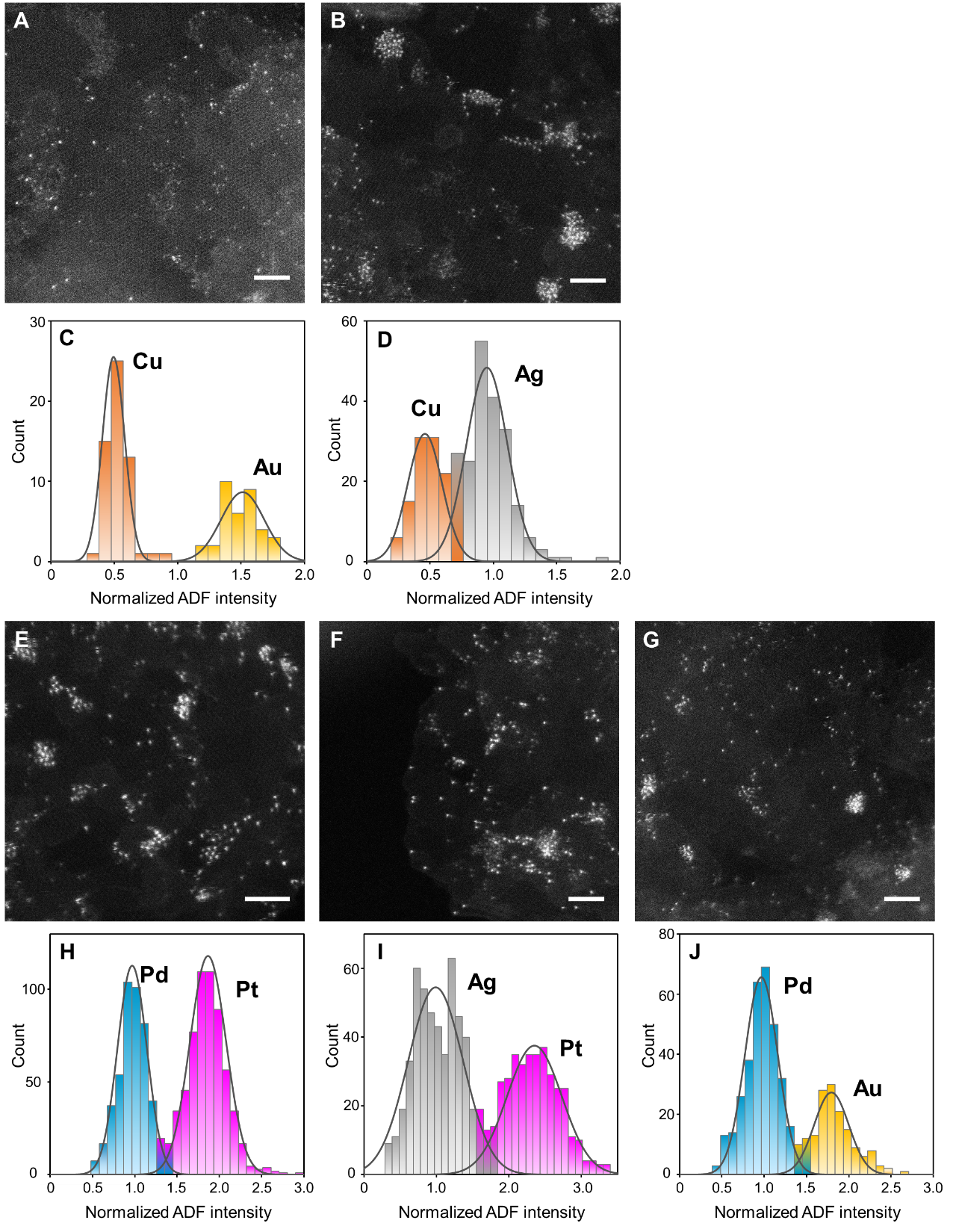


**Supplementary Figure 5.** Atomic identification for different combinations of elements. (A, B, E-G) ADF-STEM images of Au-Cu(A), Ag-Cu(B), Pt-Pd(E), Pt-Ag(F) and Au-Pd(G) bimetallic samples. Scale bar: 2 nm. (C, D, H-J) Histograms of the normalized ADF intensities extracted from the images, Au-Cu(C), Ag-Cu(D), Pt-Pd(H), Pt-Ag(I), Au-Pd(J). For the histogram of Au-Pd and Pt-Pd, the ADF intensity of Pd was normalized to 1.


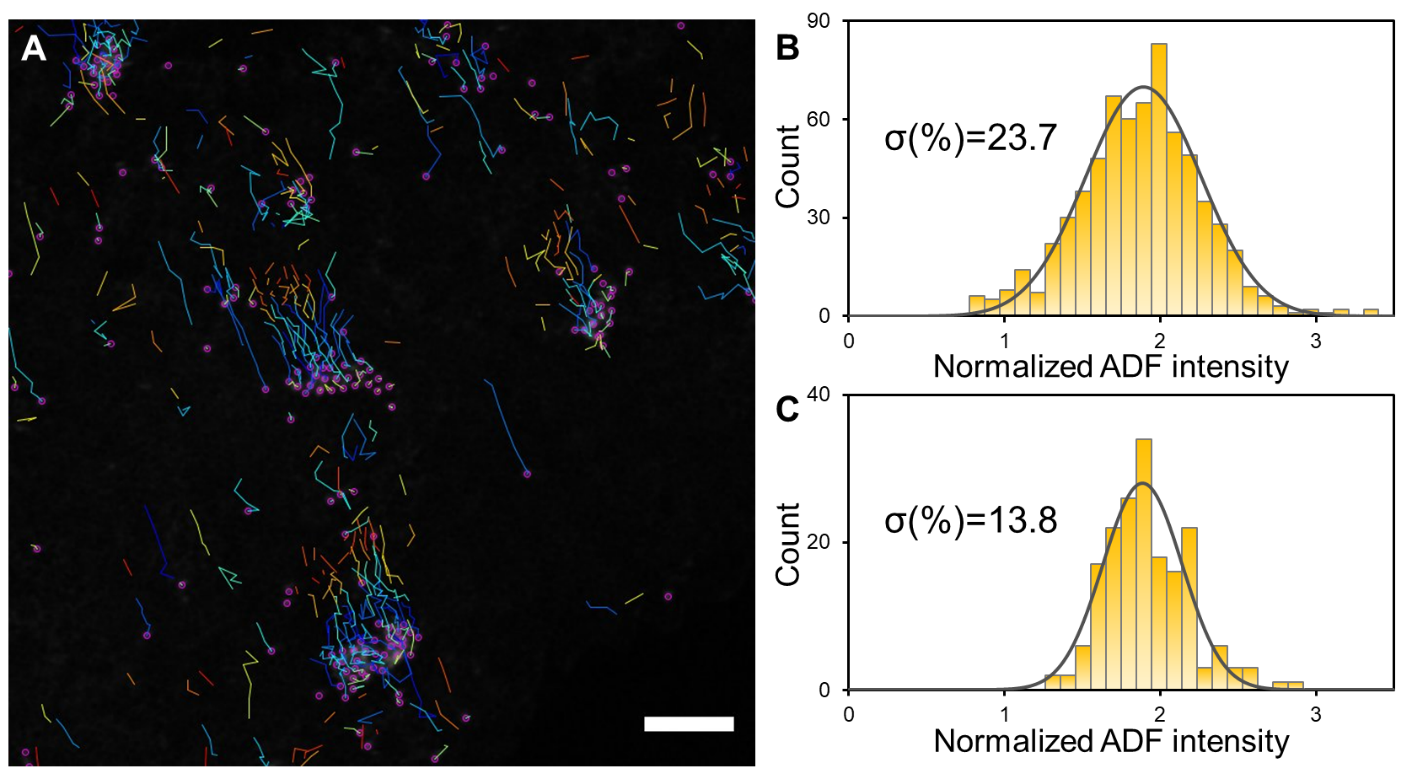


**Supplementary Figure 6.** Atom-tracking analysis of Au monometallic sample for the reduction of the standard deviation of ADF intensity. (A) The first frame of an ADF-STEM video overlayed with the tracked trajectories of Au atoms in the successive frames. Scale bar: 2 nm. (B) A histogram of normalized ADF intensity of atoms without the atom-tracking. All atoms were extracted from every 10 STEM image and values were merged together, (C) A histogram with the atom-tracking: Integrated ADF intensities of each atom based on the atom tracking over the 10 STEM images.


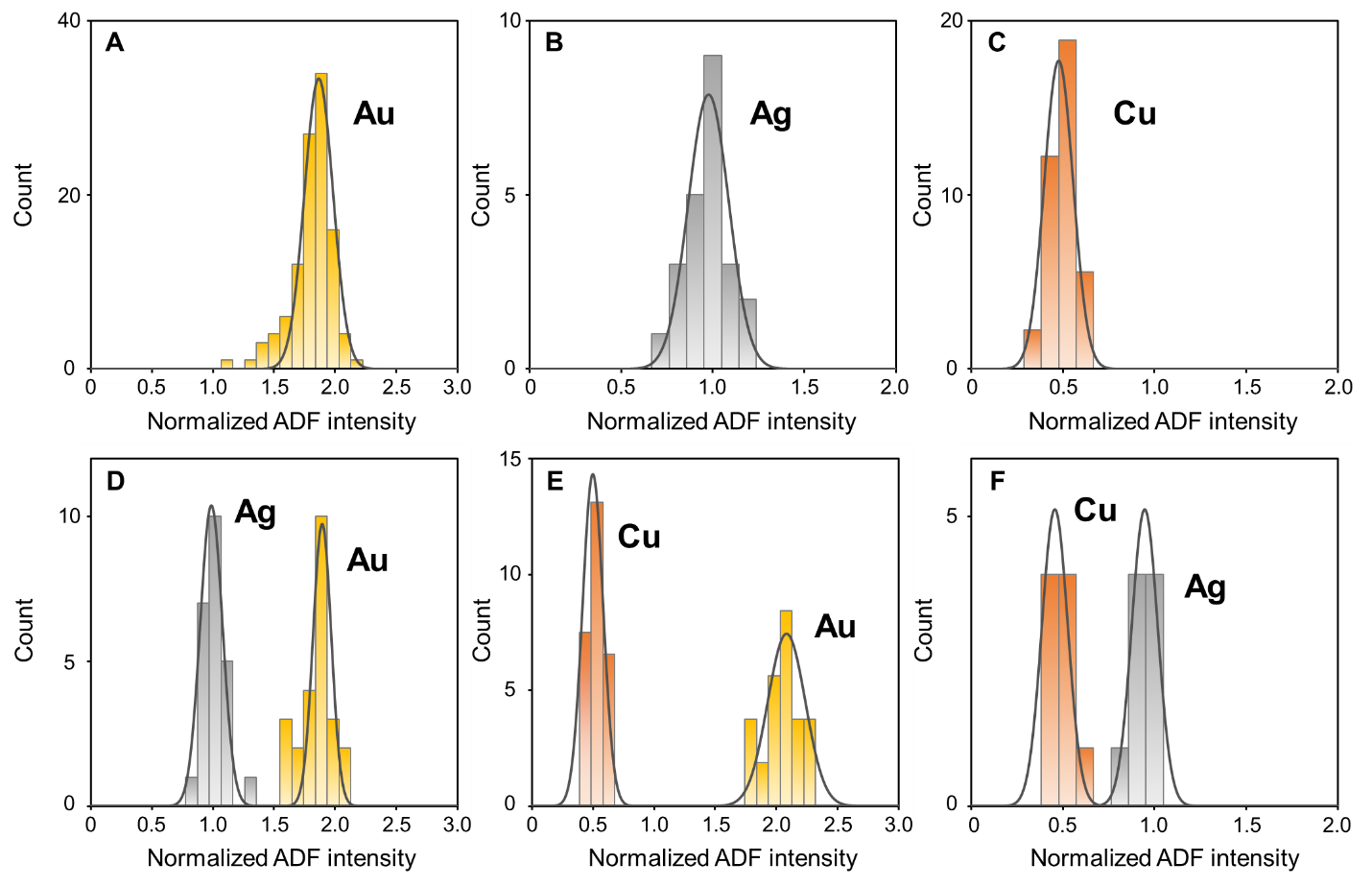


**Supplementary Figure 7.** Elemental identification of moving atoms using the atom tracking. The panels A-F correspond to (A) Au-Au, (B) Ag-Ag, (C) Cu-Cu, (D) Au-Ag, (E) Au-Cu, (F) Ag-Cu, respectively. Each histogram was created using the images in Supplementary Movies. The intensities in two adjacent frames of the STEM images were integrated.


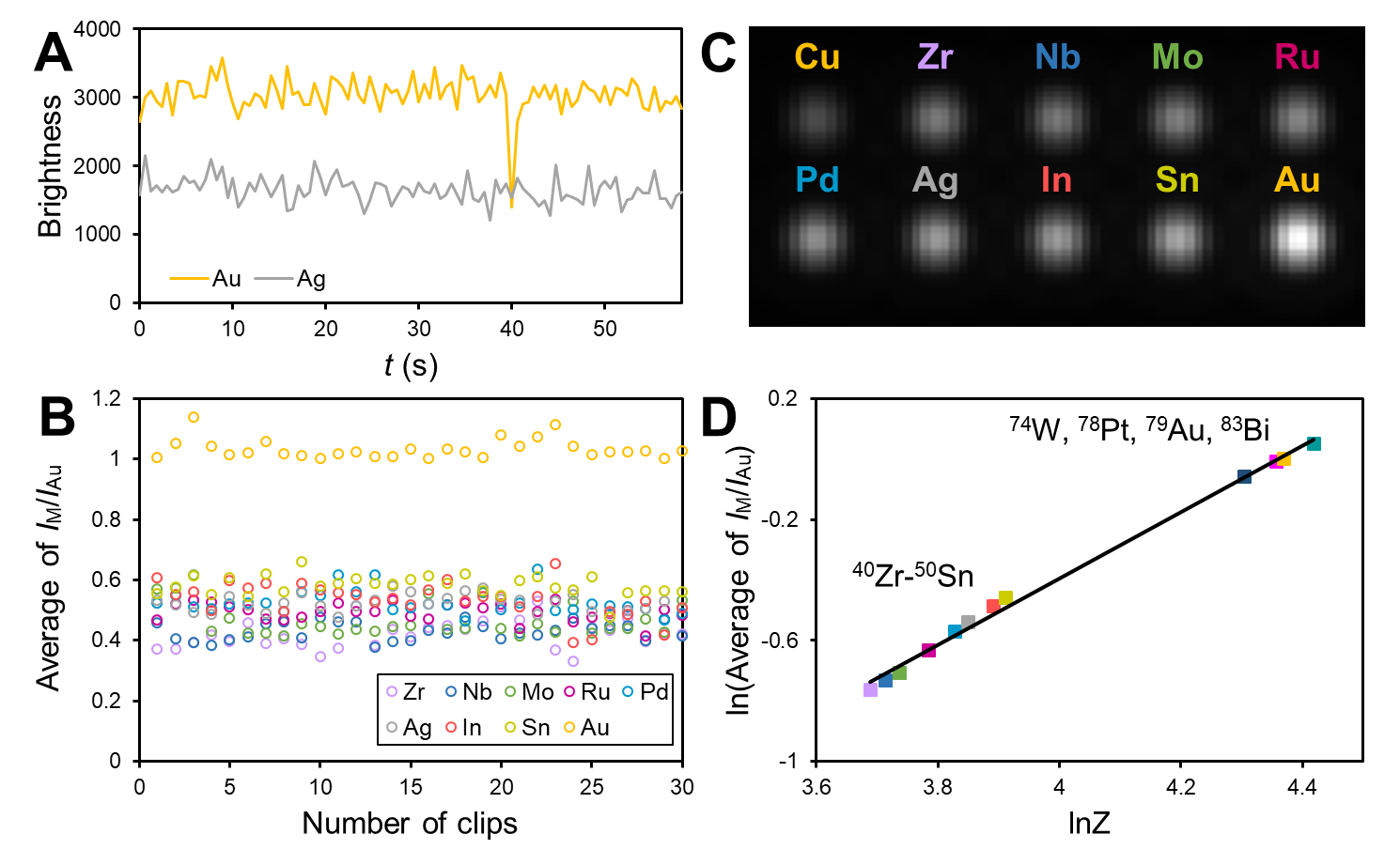


**Supplementary Figure 8.** Elemental identification of moving atoms using the atom tracking for Au- M (M=Zr, Nb, Mo, Ru, Pd, Ag, In, Sn, Au) dimers. (A) Temporal changes of the background-subtracted brightness values of two tracked atoms in an Au-Ag dimer. The atom with higher averaged brightness value is assigned to Au atom. (B) The averages of the relative brightness values (*I*_M_/*I*_Au_) obtained by the tracking analysis as in (A) with various Au-M dimers. For each Au-M combinations, at least 30 independent dimers in different fields of view were recorded as respective video clips. Each data point in this panel corresponds to the result of tracking analysis of each clip. (C) An image of various single atoms (Zr, Nb, Mo, Ru, Pd, Ag, In, Sn, W, Pt, Au and Bi) based on the ADF-STEM simulation (Supplementary Note 4). (D) The atomic number (*Z*) dependence of the relative brightness values (*I*_M_/*I*_Au_) of (C).


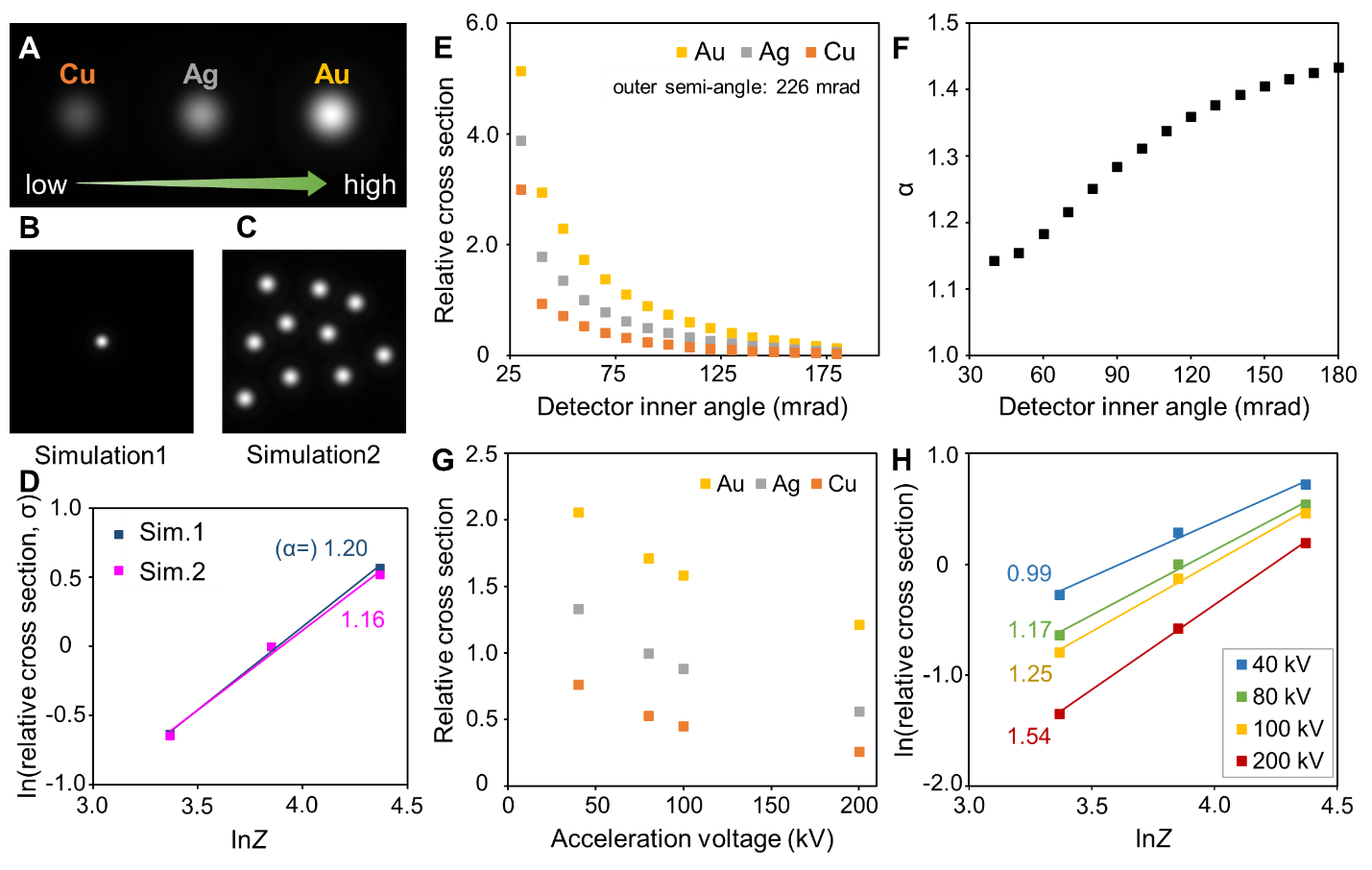


**Supplementary Figure 9.** Relationship between ADF-STEM condition and the ADF intensities. (A) A simulated image (Supplementary Note 4) of three atoms of Au, Ag and Cu on a same z-plane. (B) A simulated image of a single atom of Au. (C) A simulated image of 10 Au atoms randomly arranged on a same z-plane. (D) The logarithmic plot of *Z* (Cu, Ag and Au) versus the calculated relative cross section of single atom* based on the simulation of a single atom as the panel B (blue) and 10 atoms as the panel C (magenta). (E) The relationship between the detector inner semi-angle and the calculated relative cross section*. (F) The relationship between the detector inner semi-angle and the power order a, representing the slope in the double logarithmic plot of *Z* vs the calculated relative cross section* of single atom. (G) The relationship between acceleration voltage and the calculated relative cross section of single atom for different atoms. The detector angle was fixed from 57 mrad (inner) to 226 mrad (outer). (H) The double logarithmic plot of *Z* and the calculated relative cross section of single atom for different acceleration voltages.

*The “relative cross section of a single atom” is normalized value to an Ag atom calculated for the detector inner semi-angle of 57 mrad, the outer semi-angle of 226 mrad and acceleration voltage of 80 kV.

**
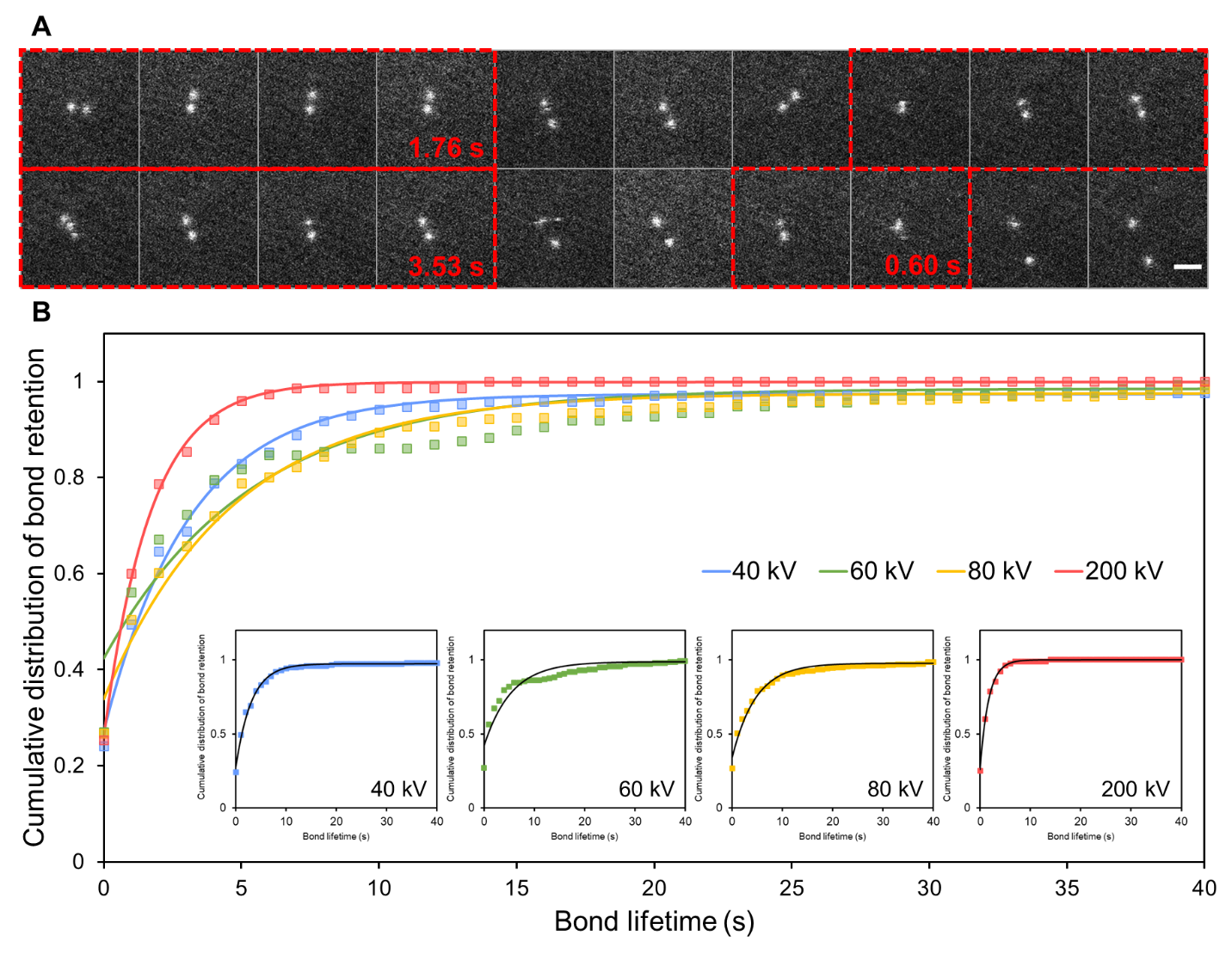
**

**Supplementary Figure 10.** Estimation of the driving force for the bond-cleavage of an Au dimer based on evaluation of the bond lifetime. (A) Images of the Au-Au dimer from the video under an electron beam scan. The acceleration voltage and the beam current were 80 kV and ca. 15 µA, respectively. Each box size is 128 ×128 pixels, and the scale bar: 0.5 nm. The interval time between the frames is about 0.6 second. The frames in three dashed boxes correspond to the bond retention times when the threshold was set to 0.30 nm, whereas the others are dissociating to two atoms. (B) The cumulative distribution of the Au-Au bond retention time (square). The horizontal axis means the retention time of Au dimers until the bond-cleavage which was defined as the moment when the bond distance exceeded the threshold of 0.30 nm. The solid line is the regression analysis result using the exponential function. The four curves correspond to the experimental results at acceleration voltages of 40 kV (blue), 60 kV (green), 80 kV (yellow), and 200 kV (red), respectively. The inset shows the results for each acceleration voltage separately.


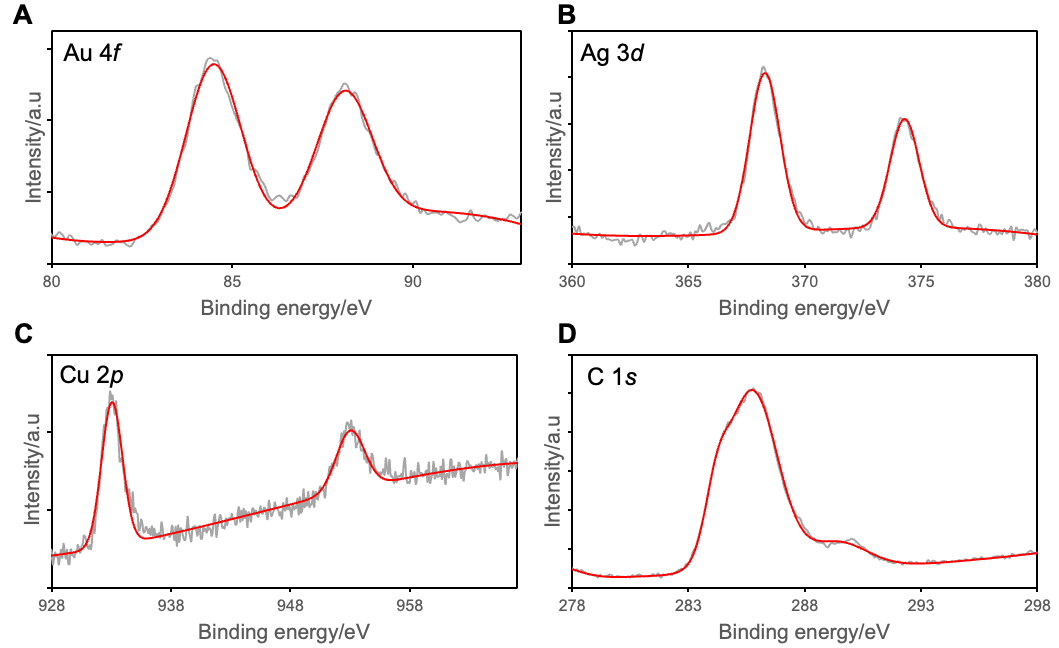


**Supplementary Figure 11.** XPS of Au-Ag-Cu trimetallic dispersion. (A) Au4*f*_5/2_ and Au4*f*_7/2_ peaks, (B) Ag3*d*_3/2_ and Ag3*d*_5/2_ peaks, (C) Cu2*p*_1/2_ and Cu2*p*_3/2_ peaks, (D) C1*s* peak.


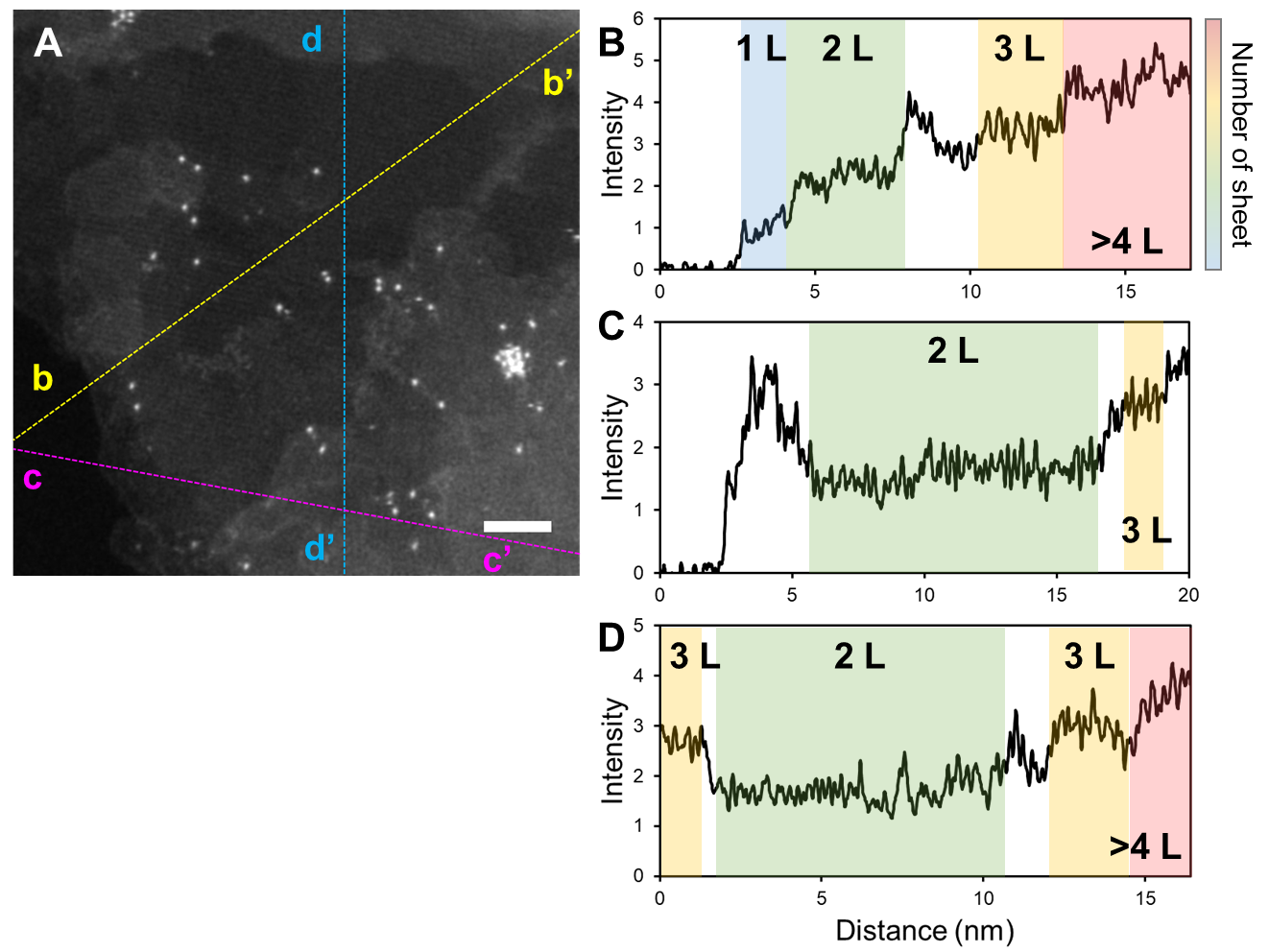


**Supplementary Figure 12.** Estimation of the thickness of a graphene sheet based on the ADF intensity. (A) An ADF-STEM image of graphene sheet with Au atoms. The three dotted lines (b-b’, c-c’ and d-d’) correspond to the cross-sectional line plots in B, C and D, respectively. A gaussian filter was applied and, the scale bar: 2 nm. (B-D) The thickness estimations on the cross-sectional line plots. The thinnest part on the dotted line A assumed to be a single layer of graphene.


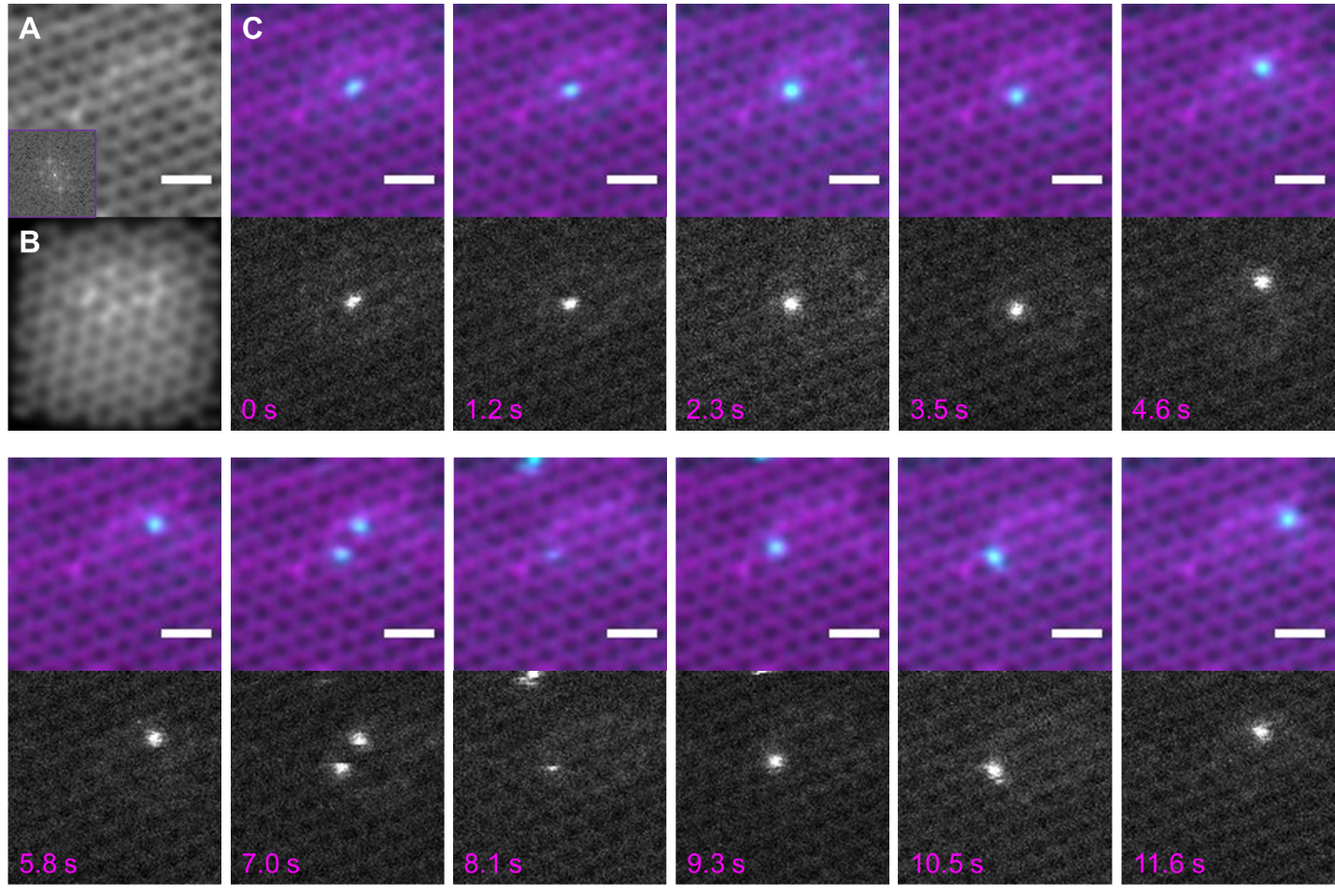


**Supplementary Figure 13.** Visualization of a graphene structure, and the trajectories of single gold atom on the graphene recorded by ADF-STEM observation. (A) Averaged STEM image (10 frames) of a graphene at the same area after the recording of the images in (C). Ten STEM images were used for average image, and a gaussian filter was applied. Scale bar: 0.5 nm. (inset) An FFT image of image (A). Six speckles appeared, indicating that it has a periodic honeycomb structure. (B) A simulated image of graphene with some carbon atoms replaced by oxygen. The oxygen atoms appear slightly brighter than the carbon atoms because of their higher atomic number. (C) The lower images represent the original images of respective frames. The upper images are composites of the image of panel A and the corresponding lower images.


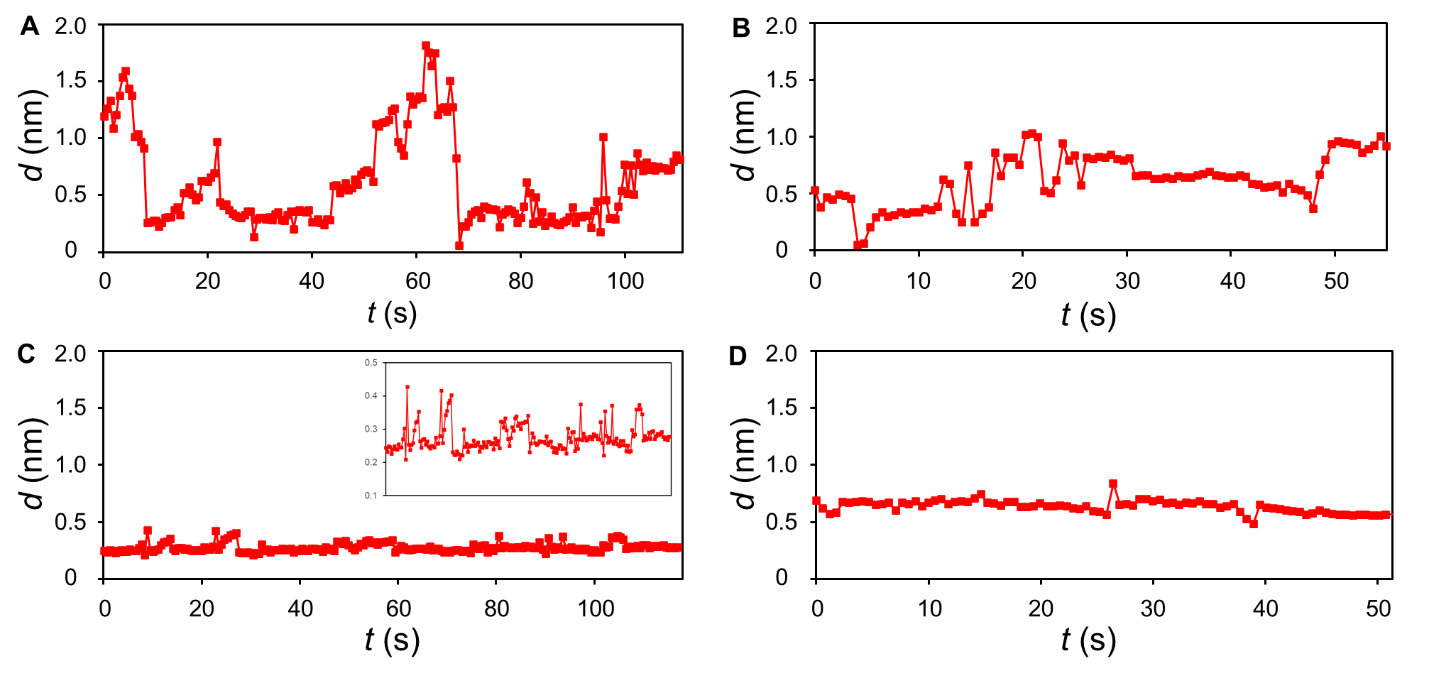


**Supplementary Figure 14.** Temporal changes of interatomic distance between two Au atoms observed at different sites. (A) Reversible formation of a dimer and two single atoms. (B) Two gold atoms formed bonding as dimer, but its bond cleavages in the middle and did not rebind. (C) Two gold atoms exist as a dimer for a long time, although the bond is stretched and contracted. (D) They remain as two single atoms for a long time without forming a bond. It should be noted, however, that the changes over time in (A) through (D) represent only a few typical examples of the large amount of data collected, 12,470 frames of images obtained from a total of 105 videos.


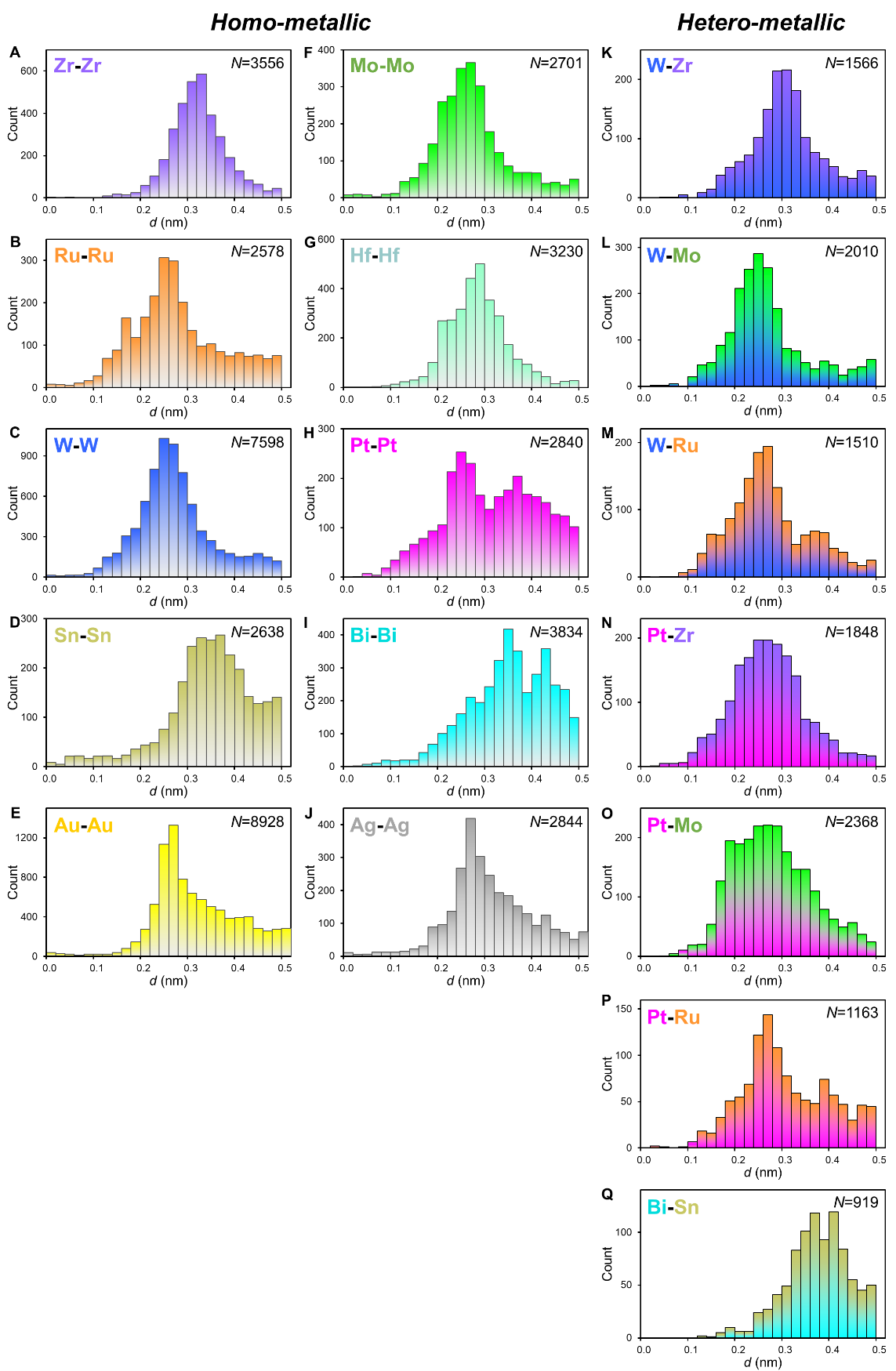


**Supplementary Figure 15.** Histograms of projected interatomic distance in various homometallic dimers (A-J) and hetero-metallic dimers (K-Q). (A-J) Respective panels correspond to Zr-Zr, Ru-Ru, W-W, Sn-Sn, Au-Au, Mo-Mo, Hf-Hf, Pt-Pt, Bi-Bi, Ag-Ag dimers. (K-Q) Respective panels correspond to W-Zr, W-Mo, W-Ru, Pt-Zr, Pt-Mo, Pt-Ru, Bi-Sn dimers.


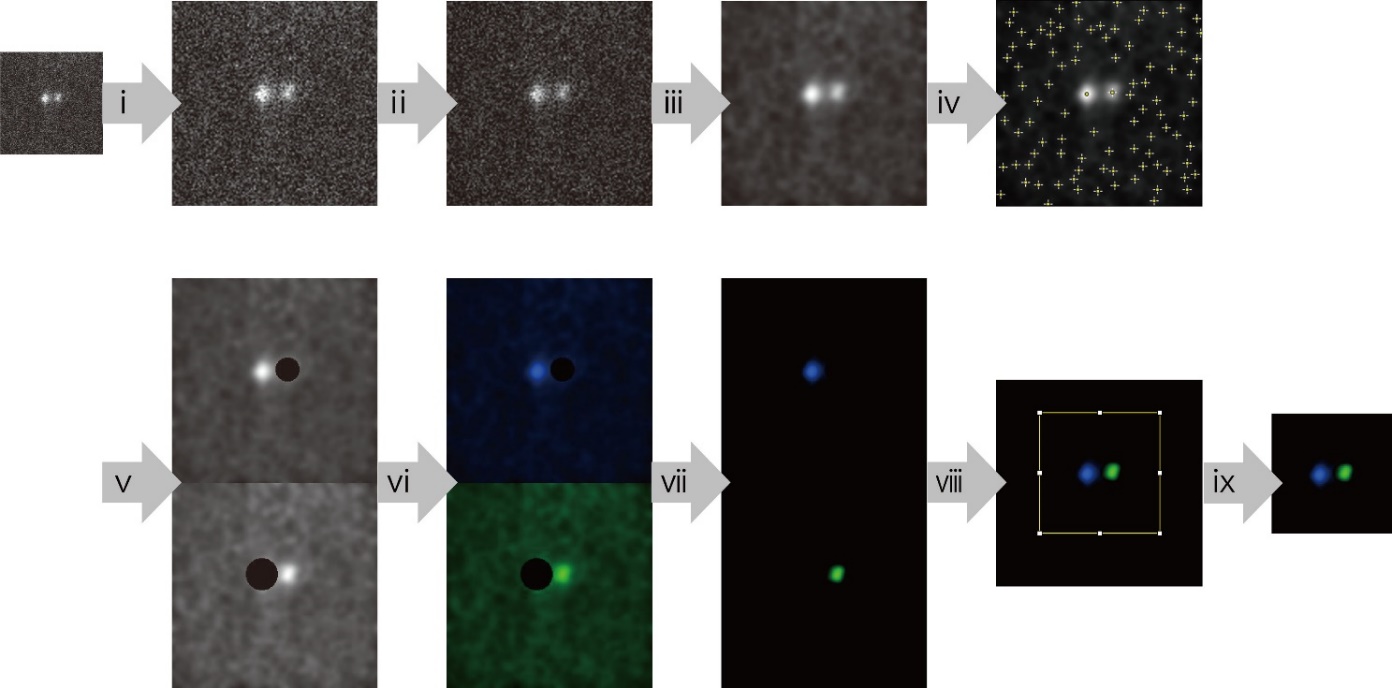


**Supplementary Figure 16.** A procedure for coloring an ADF-STEM images of a heterometallic dimer. (i)-(ix) in the Fig. S10 correspond to the following processes, respectively. (i) Resizing from 128 × 128 pixels to 256 × 256 with bicubic interpolation. (ii) Subtracting background. The “rolling ball radius” parameter was set to 50 pixels. (iii) Filtering with Gaussian filter in 4.0 pixels. (iv) Selecting the coordination for each metal atom by using Find Maxima function in 100 prominence without edge maxima. (v) Duplicating the image and deleting the brightness of another atom. (vi) Coloring for each atom. W: RGB (50, 100, 255), Mo: RGB (0, 255, 0). (vii) Adjusting brightness and contrast. (viii) Transforming images to stack and taking Z project (projection = Max intensity). Selecting square in the size of 150 × 150 pixels. Center of the square is the center of gravity of two atoms. (ix) Cropping from 256 × 256 pixels to 150 × 150 pixels.

**Supplementary Figure 17.** Optimized structures of model of graphenes with (a) zigzag and (b) armchair edges.

**Supplementary Figure 18.** Optimized structures of graphenes with (a) zigzag and (b) armchair edges, which are modified with epoxy group. The gray, red and white ball represent carbon, oxygen and hydrogen atoms.

**Supplementary Figure 19.** Optimized structures of graphenes with (a) zigzag and (b) armchair edges, which are modified with hydroxy group. The gray, red and white ball represent carbon, oxygen and hydrogen atoms.

**Supplementary Figure 20.** Optimized structures of Au dimers on graphenes with (a) zigzag and (b) armchair edges. The yellow, gray and white ball represent gold, carbon and hydrogen atoms.

**Supplementary Figure 21.** Optimized structures of Au dimers on graphenes with (a) zigzag and (b) armchair edges, which are modified with epoxy group. The yellow, gray, red and white ball represent gold, carbon, oxygen and hydrogen atoms.

**Supplementary Figure 22.** Optimized structures of Au dimers on graphenes with (a) zigzag and (b) armchair edges, which are modified with hydroxy group. The yellow, gray, red and white ball represent gold, carbon, oxygen and hydrogen atoms.

**3. Supplementary Tables**

**Supplementary Table 1** Calculated bond distances [Å] of AuAgCu trimer on different graphene models and in vacuum. The deviations from the results without considering graphene are shown in parentheses.

|  | Zigzag graphene | Armchair graphene | Vacuum (w/o graphene) |
| --- | --- | --- | --- |
| Au-Ag | 2.839 (-0.040) | 2.834 (-0.045) | 2.879 |
| Au-Cu | 2.481 (-0.002) | 2.479 (0.000) | 2.479 |
| Ag-Cu | 2.506 (+0.040) | 2.509 (+0.043) | 2.466 |

**Supplementary Table 2** Calculated bond distances of Au dimer. The deviations from the results without considering oxygen-functional groups and graphene are shown in parentheses and square brackets, respectively.

|  | w/ epoxy group | | |  | w/ hydroxy group | | |  | | w/o oxygen group | | |  |
| --- | --- | --- | --- | --- | --- | --- | --- | --- | --- | --- | --- | --- | --- |
| Zigzag | 2.543 | (+0.002) | [−0.004] |  | 2.541 | (0.000) | [−0.006] | |  | | 2.541 | [−0.006] | |
| Armchair | 2.541 | (0.000) | [−0.006] |  | 2.539 | (−0.002) | [−0.008] | |  | | 2.541 | [−0.006] | |

**4. Supplementary References**

1. Hofer, C. *et al.* Direct imaging of light-element impurities in graphene reveals triple-coordinated oxygen. *Nat. Commun.* **10**, (2019).

2. Varns, R. & Strange, P. Stability of gold atoms and dimers adsorbed on graphene. *J. Phys. Condens. Matter* **20**, (2008).

3. Chan, K. T., Neaton, J. B. & Cohen, M. L. First-principles study of metal adatom adsorption on graphene. *Phys. Rev. B - Condens. Matter Mater. Phys.* **77**, 1–12 (2008).

4. Batson, P. E. Motion of gold atoms on carbon in the aberration-corrected STEM. *Microsc. Microanal.* **14**, 89–97 (2008).

5. Furnival, T. *et al.* Anomalous diffusion of single metal atoms on a graphene oxide support. *Chem. Phys. Lett.* **683**, 370–374 (2017).

6. Kotakoski, J., Mangler, C. & Meyer, J. C. Imaging atomic-level random walk of a point defect in graphene. *Nat. Commun.* **5**, 2–6 (2014).

7. Tripathi, M. *et al.* Electron-Beam Manipulation of Silicon Dopants in Graphene. *Nano Lett.* **18**, 5319–5323 (2018).

8. Robertson, A. W. *et al.* Spatial control of defect creation in graphene at the nanoscale. *Nat. Commun.* **3**, (2012).

9. Warner, J. H. *et al.* Structural transformations in graphene studied with high spatial and temporal resolution. *Nat. Nanotechnol.* **4**, 500–504 (2009).

10. Meyer, J. C. *et al.* Accurate measurement of electron beam induced displacement cross sections for single-layer graphene. *Phys. Rev. Lett.* **108**, 1–6 (2012).

11. Chai, J. Da & Head-Gordon, M. Long-range corrected hybrid density functionals with damped atom-atom dispersion corrections. *Phys. Chem. Chem. Phys.* **10**, 6615–6620 (2008).
