# Supplementary material for: Dynamic hetero-metallic bondings visualized by sequential atom imaging
Source: Nat Commun. 2022 May 27;13:2968. doi: 10.1038/s41467-022-30533-y (PMC9142510; doi:10.1038/s41467-022-30533-y)
Supplement: Supplementary file 2 — Description of Additional Supplementary Information [file 41467_2022_30533_MOESM2_ESM.pdf]

Description of Additional Supplementary Files

File Name: Supplementary Movie 1
Description: HAADF-STEM movies showing the random movement of a single Au atom under electron beams irradiation. The three movies from left to right correspond to raw image, preprocessed image (noise reduction and background subtraction), and its jet colormap display, respectively. The box size is 2.5 × 2.5 nm with 128 ×128 pixels, and the frame intervals are about 0.6 s.

File Name: Supplementary Movie 2
Description: HAADF-STEM movies of homometallic and heterometallic dimers based on Au, Ag and Cu. All the movie clips are raw images of six dimers. The recording conditions are the same as those in Supplementary Movie 1.

File Name: Supplementary Movie 3
Description: HAADF-STEM movies of homometallic and heterometallic dimers based on Au, Ag and Cu. The video shows preprocessed images (noise reduction and background subtraction) of the six movie clips in Supplementary Movie 2.

File Name: Supplementary Movie 4
Description: Supplementary Movie 4 HAADF-STEM movies of homometallic and heterometallic dimers based on Au, Ag and Cu. The video shows jet colormap display of the six movie clips in Supplementary Movie 3.

File Name: Supplementary Movie 5
Description: Movies of the homo- and heterometallic dimers shown in Fig. 5D and 5E. The three movies correspond to raw data (top), the coloring image sequence by the element based on the intensity value (middle), and the overlay images of upper and middle movies (bottom), respectively. The images shown in Fig. 5D and 5E are both one frame of this movie. The actual size of one side of each frame is 1.22 nm.
